# An Enzyme with High Catalytic Proficiency Utilizes Distal Site Substrate Binding Energy to Stabilize the Closed State but at the Expense of Substrate Inhibition

**DOI:** 10.1021/acscatal.1c05524

**Published:** 2022-02-22

**Authors:** Angus J. Robertson, F. Aaron Cruz-Navarrete, Henry P. Wood, Nikita Vekaria, Andrea M. Hounslow, Claudine Bisson, Matthew J. Cliff, Nicola J. Baxter, Jonathan P. Waltho

**Affiliations:** † School of Biosciences, The University of Sheffield, Sheffield, S10 2TN, United Kingdom; ‡ Manchester Institute of Biotechnology and Department of Chemistry, The University of Manchester, Manchester, M1 7DN, United Kingdom

**Keywords:** Enzyme catalytic proficiency, Phosphoryl transfer mechanism, Transition state analogue, X-ray crystallography, NMR spectroscopy

## Abstract

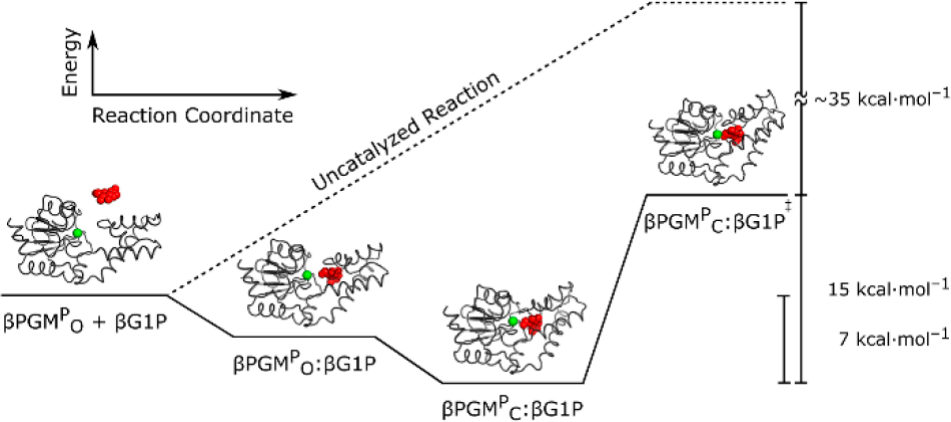

Understanding the factors that underpin the enormous catalytic proficiencies of enzymes is fundamental to catalysis and enzyme design. Enzymes are, in part, able to achieve high catalytic proficiencies by utilizing the binding energy derived from nonreacting portions of the substrate. In particular, enzymes with substrates containing a nonreacting phosphodianion group coordinated in a distal site have been suggested to exploit this binding energy primarily to facilitate a conformational change from an open inactive form to a closed active form, rather than to either induce ground state destabilization or stabilize the transition state. However, detailed structural evidence for the model is limited. Here, we use β-phosphoglucomutase (βPGM) to investigate the relationship between binding a phosphodianion group in a distal site, the adoption of a closed enzyme form, and catalytic proficiency. βPGM catalyzes the isomerization of β-glucose 1-phosphate to glucose 6-phosphate via phosphoryl transfer reactions in the proximal site, while coordinating a phosphodianion group of the substrate(s) in a distal site. βPGM has one of the largest catalytic proficiencies measured and undergoes significant domain closure during its catalytic cycle. We find that side chain substitution at the distal site results in decreased substrate binding that destabilizes the closed active form but is not sufficient to preclude the adoption of a fully closed, near-transition state conformation. Furthermore, we reveal that binding of a phosphodianion group in the distal site stimulates domain closure even in the absence of a transferring phosphoryl group in the proximal site, explaining the previously reported β-glucose 1-phosphate inhibition. Finally, our results support a trend whereby enzymes with high catalytic proficiencies involving phosphorylated substrates exhibit a greater requirement to stabilize the closed active form.

## Introduction

The ability of enzymes to achieve enormous catalytic proficiencies remains the subject of intense investigation, leading to continual progress in understanding enzyme active site electronics, structure, and dynamics. Electrostatic stabilization of the chemical transition state,^1,2^ ground state destabilization,^3−5^ efficient formation of near-attack conformers in the ground state,^6^ and contributions from conformational motions^7−9^ are all argued to contribute to catalytic proficiency. Additionally, stabilizing interactions between the enzyme active site and nonreacting portions of the substrate^3^ are also thought to play an important role. Hexokinase, for example, can catalyze phosphoryl transfer from ATP to glucose 4 × 10^4^-fold faster than from ATP to water, and this rate acceleration was ascribed to interactions with portions of glucose that do not participate in the catalytic step, rather than differences in the chemical reactivity of the two substrates.^3,10^ An analysis of the contribution of nonreacting parts of a substrate to enzyme catalytic proficiency was performed using the phosphoryl transfer enzyme rabbit muscle α-phosphoglucomutase (αPGM).^11,12^ Particularly, binding of the substrate phosphodianion group was found to be a major contributing factor, where a 3 × 10^4^-fold acceleration in phosphoryl transfer rate from phosphorylated αPGM to xylose was observed when inorganic phosphite (HPO_3_^2–^) was bound simultaneously in the active site. More recently, studies on the importance of binding a nonreacting phosphodianion group in a distal site to enhance catalytic proficiency have focused on glycerol 3-phosphate dehydrogenase (GPDH), orotidine 5′-monophosphate decarboxylase (OMPDC), and triose phosphate isomerase (TIM).^13−16^ Despite the substantially different transition states stabilized by these enzymes, the interaction between the enzyme and the phosphodianion group contributes a consistent 11–13 kcal·mol^–1^ reduction in the activation energy barrier for their reactions.^13−15,17^ In each of these enzymes, a phosphodianion group is held in a positively charged distal site, and kinetic studies have shown that 50–80% of the intrinsic binding energy is provided through interactions with either a single arginine residue in GPDH and OMPDC or a lysine residue in TIM.^18−20^

In general, enhanced catalytic proficiency usually involves sequestration of the substrate(s) in a low dielectric environment, coordinated extensively by a network of electrostatic interactions between active site residues, cofactors, and specific water molecules within a closed active form.^1^ Enzyme conformational changes required to achieve this closed form can range from large domain movements to subtle rearrangements of flexible loops. In a phosphodianion-driven enzyme-activation framework,^21−24^ the energy derived from the binding of a phosphodianion group in a distal site is used to facilitate substrate sequestration, rather than to promote catalysis through ground state destabilization.^3−5^ However, if the utilization of this energy is perturbed by a distal site mutation, then the lowest free energy enzyme–substrate complex conformation populated in the reaction coordinate (i.e., the Michaelis complex) can change from a closed active form (E_C_:S) to an open inactive form (E_O_:S). In this scenario, adoption of the closed active form (E_O_:S → E_C_:S) becomes part of the rate-limiting process of the reaction. An underlying assumption of this framework, which remains to be fully tested experimentally, is that the binding energy of the phosphodianion group in the distal site does not also specifically reduce the transition state energy barrier for the chemical step.^24^ Simulations have been used to support this assumption, and they suggest that E_C_:S is equally reactive, regardless of the presence or absence of the substrate phosphodianion group.^23^ Hence, the intrinsic binding energy of the phosphodianion group only stabilizes the transition state indirectly, through facilitating the adoption of E_C_:S; i.e., the phosphodianion group behaves as a spectator during the chemical step. Although this binding energy is consistent in magnitude across the three systems studied previously, the Michaelis complex is not. For GPDH and TIM (catalyzing hydride transfer and proton transfer reactions, respectively), the Michaelis complex is E_O_:S,^18,20^ and either a large domain reorientation or small loop rearrangements are observed upon the formation of E_C_:S, respectively.^21,25^ In contrast, for OMPDC (catalyzing the decarboxylation of orotidine 5′-monophosphate via a vinyl carbanion intermediate), the Michaelis complex is E_C_:S,^19^ where widespread conformational changes involving several loops are required to achieve the closed enzyme form.^26^ Therefore, the identity of the Michaelis complex does not appear to correlate with the magnitude of the conformational changes needed for the adoption of the closed active form.

**Figure 1:**
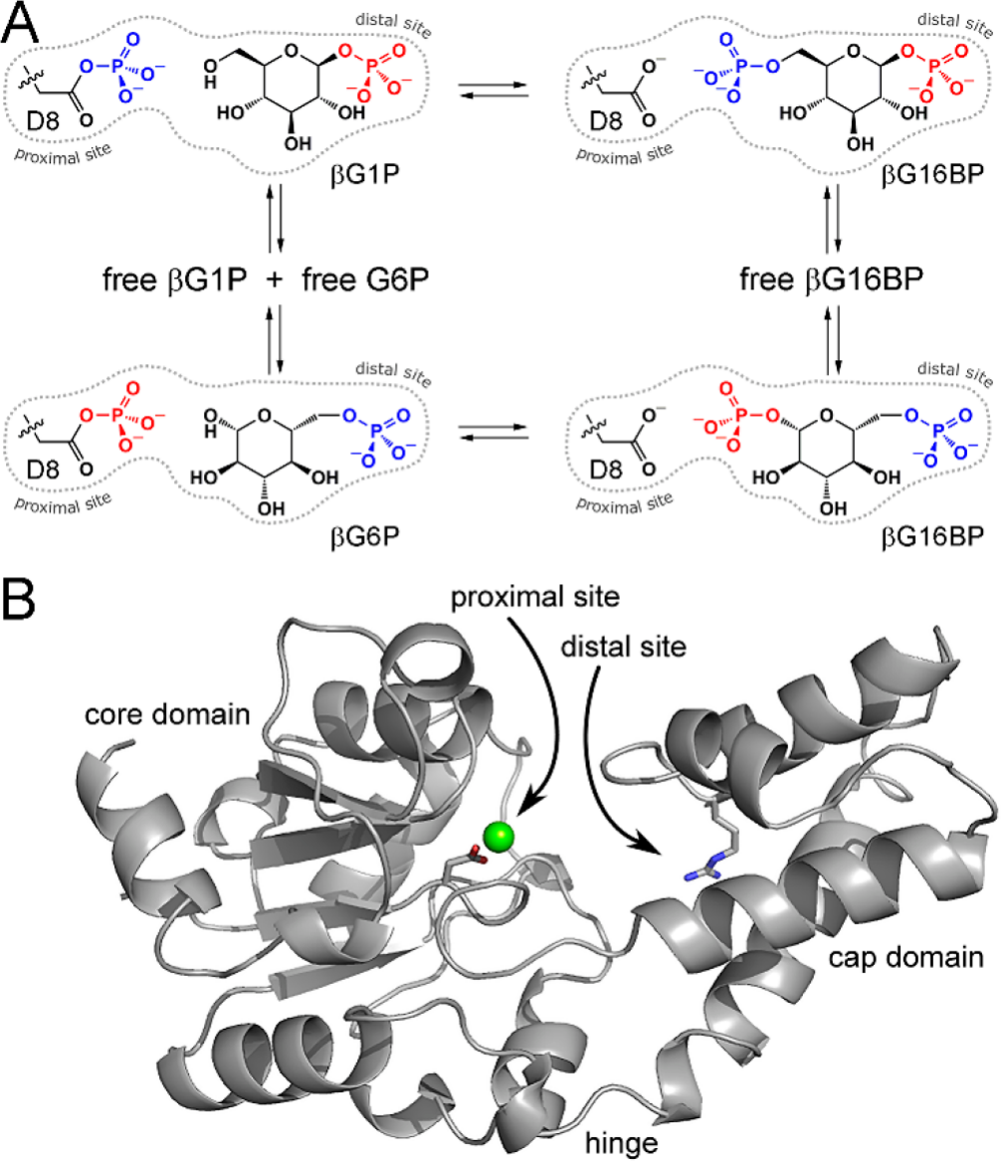
βPGM catalytic cycle and enzyme architecture. (A) βPGM catalytic cycle for the enzymatic conversion of βG1P to G6P via a βG16BP reaction intermediate. The phosphoryl transfer reaction between the phospho-enzyme (βPGM^P^, phosphorylated at residue D8) and βG1P is illustrated with the transferring phosphate (blue) in the proximal site and the phosphodianion group (red) of βG1P in the distal site. βG16BP is released to solution, which subsequently rebinds in the alternative orientation.^29^ Here, the phosphoryl transfer reaction between βPGM and βG16BP is shown with the transferring phosphate (red) in the proximal site and the phosphodianion group (blue) of βG16BP in the distal site. G6P is released as a product, together with the regeneration of βPGM^P^. (B) Cartoon representation of the substrate-free βPGM_WT_ crystal structure (PDB 6YDL)^28^ highlighting the architecture of the helical cap domain (T16–V87) and the α/β core domain (M1–D15, S88–K221). The proximal and distal phosphodianion group binding sites are located in the cleft formed between the domains, and rotation at the hinge results in closure of the active site during catalysis. Mg_cat_^2+^ (green sphere) is located in the proximal site adjacent to residue D8 (sticks), and residue R49 (sticks) coordinates the phosphodianion group of the substrate (or reaction intermediate) in the distal site.

Phosphoryl transfer enzymes are another valuable model system to further explore the relationship between catalytic proficiency, the identity of the Michaelis complex, and the degree of conformational change required during a catalytic cycle. These enzymes can achieve catalytic rate constants of greater than 100 s^–1^, despite the corresponding spontaneous noncatalyzed rate constants being ∼10^–20^ s^–1^.^27^ Among phosphoryl transfer enzymes, phosphomutases (e.g., rabbit muscle αPGM) are most appropriate for such investigations, as they not only catalyze phosphoryl transfer between the donor and acceptor groups in the proximal site but also coordinate a phosphodianion group of the substrate(s) in a distal site. In contrast to rabbit muscle αPGM, β-phosphoglucomutase (βPGM, EC 5.4.2.6, 25 kDa) from *Lactococcus lactis* is a HAD superfamily phosphomutase and catalyzes the reversible isomerization of β-glucose 1-phosphate (βG1P) to glucose 6-phosphate (G6P) via a β-glucose 1,6-bisphosphate intermediate (βG16BP) with a catalytic proficiency of 4 × 10^26^ M^–1^ (Figure 1A).^27−38^ Substrate-free βPGM adopts an open conformation where the active site cleft, located between the cap and core domains, is exposed to bulk solvent (Figure 1B).^28,31,35^ A cap domain rotation of 33–36° at the interdomain hinge leads to a closed transition state conformation,^31^ as revealed in transition state analogue (TSA) complexes between βPGM, metallofluoride moieties, and G6P or βG1P analogues.^35,37,38^ The βPGM:AlF_4_:G6P, βPGM:MgF_3_:G6P, βPGM:AlF_4_:βG1fluorophosphonate, and βPGM:MgF_3_:βG1fluorophosphonate TSA complexes mimic the active site organization for the phosphoryl transfer chemical step. Therefore, βPGM, GPDH, and OMPDC all require large conformational changes upon the adoption of the closed active form. The phosphodianion group of G6P, βG1P, or βG16BP in the distal site is coordinated by the guanidinium group of residue R49 in an analogous arrangement to that present between the nonreacting phosphodianion group of the corresponding substrate and the distal site cationic side chains of residue R269 in GPDH, residue R235 in OMPDC and residue K12 in TIM.^39^

A valuable property of βPGM is its amenability to analysis by a variety of NMR techniques and high-resolution X-ray crystallography,^31,32,35−38,40−42^ which allows βPGM to be used as a model system to tackle some remaining questions about how enzymes utilize the substrate binding energy to achieve high catalytic proficiency. Here, we show, through combined use of site-directed mutagenesis, kinetic assays, NMR spectroscopy, and X-ray crystallography, that perturbation of the cation-phosphodianion interaction in the distal site using the R49K (βPGM_R49K_) and R49A (βPGM_R49A_) variants of βPGM reveals that the Michaelis complex is E_C_:S for βPGM_WT_. NMR chemical shift comparisons of βPGM_R49K_:AlF_4_:G6P, βPGM_R49A_:AlF_4_:G6P, βPGM_R49K_:MgF_3_:G6P, and βPGM_R49A_:MgF_3_:G6P TSA complexes, together with their βPGM_WT_ counterparts, indicate that side chain substitution in the distal site is not sufficient to preclude the adoption of a fully closed, near-transition state conformation. These observations justify the underlying assumption of the framework where the cation–phosphodianion interaction energy is not utilized substantially in catalyzing the chemical step. Furthermore, stabilization of E_C_:S through binding of the phosphodianion group of βG1P in the distal site by substrate-free βPGM produces substrate inhibition, as demonstrated by the structural characterization of a fully closed, inhibited βPGM:βG1P complex. Significantly, the identity of the Michaelis complex along with the enormous catalytic proficiency reported aligns βPGM with OMPDC, rather than with GPDH or TIM. Therefore, these results support a trend, whereby enzymes with high catalytic proficiencies involving phosphorylated substrates primarily utilize the cation–phosphodianion interaction energy for stabilization of E_C_:S. Finally, examination of the multitude of new and previously reported crystal structures for βPGM enables a detailed illustration of the E_O_:S to E_C_:S transition.

## Results

### Structures of Substrate-Free βPGM_R49K_ and Substrate-Free βPGM_R49A_

Variants βPGM_R49K_ and βPGM_R49A_ were used to study the cationic side chain of residue R49 and its contribution to coordinating the phosphodianion group of G6P in the distal site. The solution behaviors of substrate-free βPGM_R49K_ and substrate-free βPGM_R49A_ were compared to substrate-free βPGM_WT_ using ^1^H^15^N-TROSY NMR experiments (Figure S1A,B). The near-equivalence in backbone amide chemical shifts for βPGM_R49K_ and βPGM_WT_ indicates that the substitution only impacts its immediate vicinity. In contrast, the small chemical shift perturbations in the cap domain of βPGM_R49A_ reveal that the loss of the bulky cationic side chain has an additional, subtle effect on its helical packing arrangement when compared to βPGM_WT_. *cis–trans* isomerization of the K145–P146 peptide bond previously observed in βPGM_WT_^28^ is also present in βPGM_R49K_ and βPGM_R49A_, resulting in two conformers in slow exchange (∼70% *cis*-P146 and ∼30% *trans*-P146). Substrate-free βPGM_R49K_ (1.6 Å resolution, PDB 6HDH) and substrate-free βPGM_R49A_ (2.0 Å resolution, PDB 6HDI) were crystallized, and their structures were determined (Table S1 and Figure S1C,D). Both structures overlay closely with previously deposited substrate-free βPGM_WT_ structures (PDB 1ZOL and PDB 2WHE;^31,35^Figure S2 and Figure S3), and the catalytic magnesium ion (Mg_cat_^2+^) in the proximal site is coordinated analogously. Comparisons of the distal site confirm the NMR results that there is minimal structural perturbation of residues near the substitution site in βPGM_R49K_ (Figure S1A,C) and βPGM_R49A_ (Figure S1B,D). The subtle changes in helical packing of the cap domain that are observed in the solution behavior of βPGM_R49A_ are less than the resolutions of the crystal structures. The Cβ atoms of both substituted residues K49 and A49 occupy similar positions to that of residue R49 in βPGM_WT_. In summary, only a local impact is observed in the behavior of the cap domain upon R49 side chain substitution in substrate-free βPGM_R49K_ and substrate-free βPGM_R49A_.

### Structures of the βPGM_R49K_ and βPGM_R49A_ TSA Complexes

**Figure 2:**
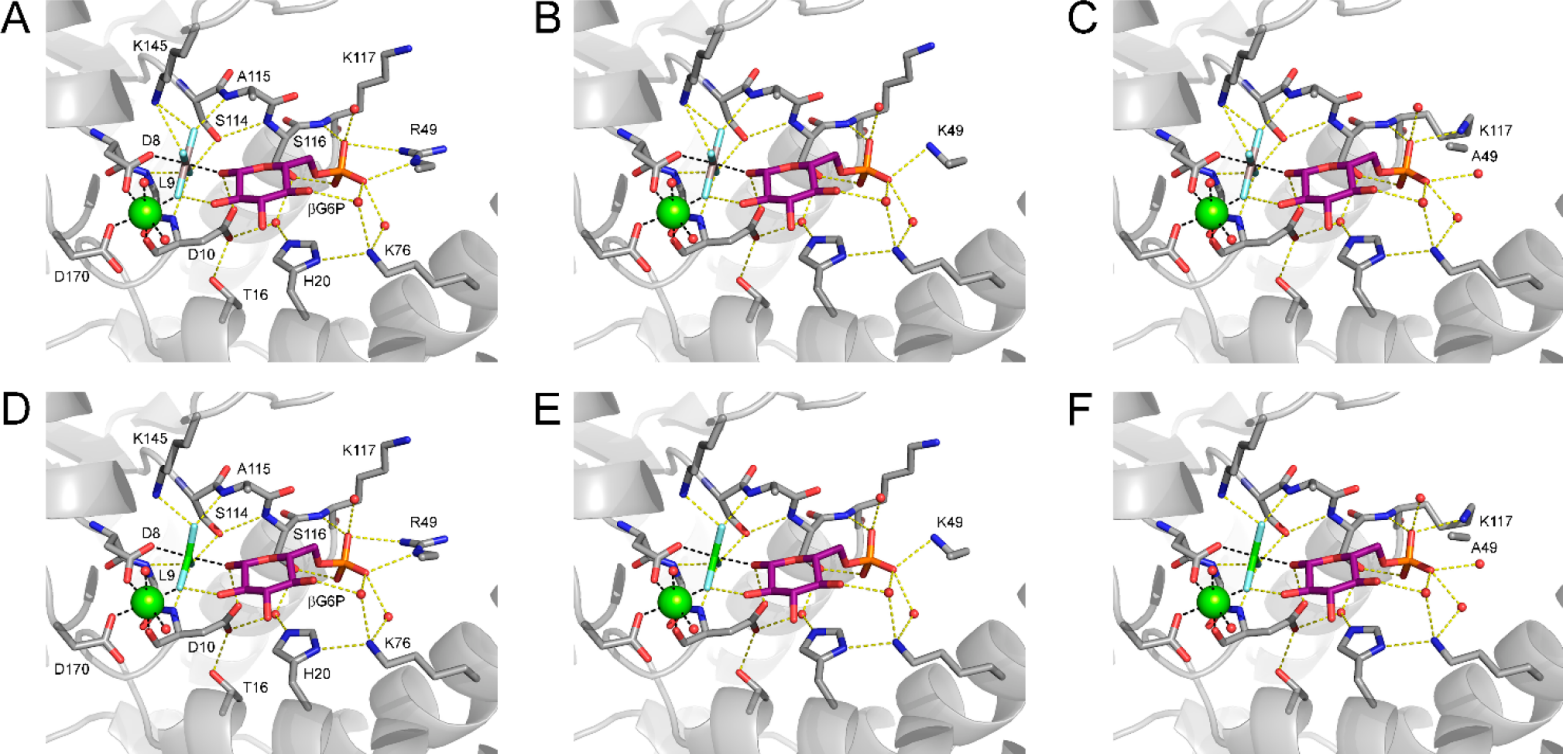
Crystal structure comparisons of the βPGM:AlF_4_:G6P and βPGM:MgF_3_:G6P TSA complexes. Active site details of (A) βPGM_WT_:AlF_4_:G6P complex (PDB 2WF6), (B) βPGM_R49K_:AlF_4_:G6P complex (PDB 6HDJ), (C) βPGM_R49A_:AlF_4_:G6P complex (PDB 6HDK), (D) βPGM_WT_:MgF_3_:G6P complex (PDB 2WF5),^35^ (E) βPGM_R49K_:MgF_3_:G6P complex (PDB 6HDL), and (F) βPGM_R49A_:MgF_3_:G6P complex (PDB 6HDM). Selected residues (sticks), together with the square-planar AlF_4_^–^ moiety (dark gray and light blue sticks), the trigonal-planar MgF_3_^–^ moiety (green and light blue sticks), βG6P (purple carbon atoms), structural waters (red spheres), and Mg_cat_^2+^ (green sphere) are illustrated. Yellow dashes indicate hydrogen bonds, and black dashes show metal ion coordination. For R49 and K49, the Cα and Cβ atoms have been omitted for clarity. The side chain of residue N118, which coordinates one of the phosphodianion oxygen atoms of G6P equivalently in the TSA complexes, has also been omitted for clarity.

Variants βPGM_R49K_ and βPGM_R49A_ were studied as their TSA complexes to investigate the contribution of the cationic side chain of R49 to the coordination of the phosphodianion group in the distal site in a fully closed, near-transition state conformation. βPGM_WT_, βPGM_R49K_, and βPGM_R49A_ were crystallized in a complex with AlF_4_^–^ and G6P using standard conditions,^35,37,40^ and the structures of the resulting βPGM_WT_:AlF_4_:G6P (1.4 Å resolution, PDB 2WF6), βPGM_R49K_:AlF_4_:G6P (1.2 Å resolution, PDB 6HDJ), and βPGM_R49A_:AlF_4_:G6P (1.2 Å resolution, PDB 6HDK) TSA complexes were obtained (Table S1). When compared with the βPGM_WT_:AlF_4_:G6P TSA complex, the βPGM_R49K_:AlF_4_:G6P and βPGM_R49A_:AlF_4_:G6P TSA complexes show equivalent full domain closure, together with near-identical domain conformations and proximal site coordination of the square-planar AlF_4_^–^ moiety (Figure 2A,B,C, Figures S2, S3, and S4A,B).

However, although the phosphodianion group of G6P is located in the same position in the distal site in each of the TSA complexes, its coordination differs between the βPGM_WT_:AlF_4_:G6P TSA complex and the βPGM_R49K_:AlF_4_:G6P and βPGM_R49A_:AlF_4_:G6P TSA complexes (Figure 2A,B,C). In the βPGM_WT_:AlF_4_:G6P TSA complex, the phosphodianion group is coordinated by the backbone amide group of K117 and the side chains of S116 and N118, together with the guanidinium side chain of residue R49 through two hydrogen bonds to separate phosphodianion oxygen atoms of G6P. In the βPGM_R49K_:AlF_4_:G6P TSA complex, the alkylammonium side chain of residue K49 is only able to hydrogen bond to one of these oxygen atoms, although the remaining coordination in the distal site is equivalent (Figure 2B and Figure S4A). In the βPGM_R49A_:AlF_4_:G6P TSA complex, the A49 side chain cannot substitute for either of the missing R49 side chain hydrogen bonding interactions that coordinate the phosphodianion oxygen atoms of G6P. Instead, the alkylammonium side chain of residue K117 located in the core domain on the opposite face of the active site is recruited into the distal site from a solvent exposed position, thereby providing a surrogate hydrogen bonding interaction between a cationic group and the phosphodianion group (Figure 2C and Figure S4B). Moreover, an additional water molecule coordinates the phosphodianion group compared to the βPGM_WT_:AlF_4_:G6P TSA complex. In conclusion, both βPGM_R49K_ and βPGM_R49A_ can adopt a fully closed, near-transition state conformation despite the local perturbation that the R49 side chain substitution imposes on the coordination of the phosphodianion group of G6P in the distal site. Furthermore, the repositioning of other side chains located in the active site offers a degree of redundancy in hydrogen bonding interactions.

Structural investigations were extended to include TSA complexes of βPGM_R49K_ and βPGM_R49A_ containing a trigonal-planar MgF_3_^–^ moiety. MgF_3_^–^ complexes are more expanded and less stable than their AlF_4_^–^ counterparts, owing to the instability of MgF_3_^–^ in solution.^32^ However, the trigonal-planar MgF_3_^–^ moiety in the proximal site is near-isosteric and isoelectronic with PO_3_^–^ and therefore is a closer mimic of the transition state for the chemical step.^35,40,43^ βPGM_R49K_ and βPGM_R49A_ were crystallized in complex with MgF_3_^–^ and G6P using conditions published previously,^28,35,37^ and the structures of the resulting βPGM_R49K_:MgF_3_:G6P (1.2 Å resolution, PDB 6HDL) and βPGM_R49A_:MgF_3_:G6P (1.3 Å resolution, PDB 6HDM) TSA complexes were obtained (Table S1). When compared with the βPGM_WT_:MgF_3_:G6P TSA complex (1.3 Å resolution, PDB 2WF5),^35^ the fully closed βPGM_R49K_:MgF_3_:G6P and βPGM_R49A_:MgF_3_:G6P TSA complexes show a near-identical correspondence in domain conformation and proximal site coordination of the trigonal-planar MgF_3_^–^ moiety (Figure 2D,E,F and Figures S2, S3, and S4C,D). Additionally, the βPGM_R49K_:MgF_3_:G6P and βPGM_R49A_:MgF_3_:G6P TSA complexes show equivalent coordination of the phosphodianion group in the distal site compared to the βPGM_R49K_:AlF_4_:G6P and βPGM_R49A_:AlF_4_:G6P TSA complexes, respectively (Figure 2 and Figure S4).

### Measurement of Apparent G6P Dissociation Constants in the βPGM_R49K_ and βPGM_R49A_ TSA Complexes

The βPGM_R49K_:AlF_4_:G6P, βPGM_R49A_:AlF_4_:G6P, βPGM_R49K_:MgF_3_:G6P, and βPGM_R49A_:MgF_3_:G6P TSA complexes were investigated further using NMR spectroscopy to examine their solution properties. All four TSA complexes readily self-assemble in solution from mixtures containing 0.5–1.5 mM βPGM, 5 mM MgCl_2_, 15 mM NaF, (3 mM AlCl_3_), and 20 mM G6P in K^+^ HEPES buffer (pH 7.2). Since the free AlF_4_^–^ anion is well-populated in solution,^44^ a βPGM:AlF_4_ complex readily forms in the absence of G6P, which represents a TSA of phospho-enzyme (βPGM^P^, phosphorylated at residue D8, Figure 1A) hydrolysis.^35,40,45^ Therefore, each apparent dissociation constant (*K*_d_) of G6P was determined by titration into separate βPGM_R49K_:AlF_4_ and βPGM_R49A_:AlF_4_ complexes, and the formation of the βPGM_R49K_:AlF_4_:G6P and βPGM_R49A_:AlF_4_:G6P TSA complexes was monitored using one-dimensional ^1^H NMR spectra. The changing intensity of the well-resolved indole resonance of residue W24 (acting as a reporter for G6P binding and adoption of the closed TSA complex in slow exchange) was fitted to determine apparent *K*_d_ (G6P) values for the βPGM_R49K_:AlF_4_:G6P TSA complex (apparent *K*_d_ (G6P) = 3.0 ± 0.4 mM) and the βPGM_R49A_:AlF_4_:G6P TSA complex (apparent *K*_d_ (G6P) = 18 ± 1 mM; and Figure S5A,B). For the βPGM_WT_:AlF_4_:G6P TSA complex, an apparent *K*_d_ (G6P) = 9 ± 1 μM (Table 1) was determined using isothermal titration calorimetry (as the apparent *K*_d_ (G6P) is too low to be resolved by NMR methods).^35^ An equivalent NMR approach to determine apparent *K*_d_ (G6P) values for the βPGM_R49K_:MgF_3_:G6P and βPGM_R49A_:MgF_3_:G6P TSA complexes was not used because the formation constant for MgF_3_^–^ in solution is very low, and βPGM_R49K_:MgF_3_ and βPGM_R49A_:MgF_3_ complexes are not detectable.^40^ Compared to the βPGM_WT_:AlF_4_:G6P TSA complex, the increases in apparent *K*_d_ (G6P) values of 330-fold and 2000-fold for the βPGM_R49K_:AlF_4_:G6P and βPGM_R49A_:AlF_4_:G6P TSA complexes, respectively, indicate that R49 side chain substitution in the distal site impacts the stability of the corresponding TSA complexes.

**Table 1:** Kinetic Parameters, Apparent *K*_d_ (G6P) (μM), *k*_obs_ (s^–1^), and *k*_cat_/*K*_m_ Ratios (s^–1^·μM^–1^) Determined for βPGM_WT_, βPGM_R49K_, and βPGM_R49A_, along with the Free Energy Changes (kcal·mol^–1^) Resulting from R49 Side Chain Substitution

enzyme	apparent *K*_d_ (G6P)	*k*_obs_	*k*_cat_/*K*_m_	ΔΔ*G*_S_^a^	ΔΔ*G*^‡^^b^	ΔΔ*G*^c^
βPGM_WT_	9 ± 1	70 ± 1	0.29	N/A	N/A	N/A
βPGM_R49K_	3000 ± 400	14.8 ± 1	0.05	3.4	0.9	4.3
βPGM_R49A_	18000 ± 1000	5.9 ± 0.5	0.02	4.5	1.5	6.0

a The free energy change in the stability of the Michaelis complex is calculated as ΔΔ*G*_S_ = *RT* ln(apparent *K*_d_(βPGM_X_)/apparent *K*_d_(βPGM_WT_)), where *R* is 1.987 × 10^–3^ kcal·mol^–1^·K^–1^, *T* = 298 K, and βPGM_X_ = βPGM_R49K_ or βPGM_R49A_.

b The free energy change in the stability of the transition state is calculated as ΔΔ*G*^‡^ = −*RT* ln(*k*_obs_(βPGM_X_)/*k*_obs_(βPGM_WT_)), where *R* is 1.987 × 10^–3^ kcal·mol^–1^·K^–1^, *T* = 298 K, and βPGM_X_ = βPGM_R49K_ or βPGM_R49A_.

c The total free energy change (ΔΔ*G*_S_ + ΔΔ*G*^‡^).

### Solution Behavior of the βPGM_R49K_ and βPGM_R49A_ TSA Complexes

Within the TSA complexes, any disruption of the proximal site due to perturbation of the coordination of the phosphodianion group in the distal site should be reflected in weighted ^1^H and ^15^N chemical shift changes of protein NMR resonances. In general, structural modifications arising from a single amino acid substitution result in chemical shift changes (Δδ) of 1–2 ppm for backbone amide groups within 5 Å of the substitution site, as the local electronic environment is perturbed.^46,47^ Significantly larger Δδ values report more pronounced alterations in protein conformation.^28^ Additionally, ^19^F chemical shifts are strongly perturbed by the electronic environment in the vicinity of the fluorine nuclei. Therefore, the presence of metallofluoride moieties in the proximal site provides a highly sensitive measurement of the extent of perturbation across the active site in the TSA complexes. For example, ^19^F Δδ values <1.7 ppm are observed for the fluorine nuclei when comparing βPGM_WT_:MgF_3_:G6P and βPGM_WT_:MgF_3_:glucose 6-phosphonate TSA complexes, where the methylene group of the nonhydrolyzable G6P analogue results in small changes to the electrostatic distribution within the distal site.^35^ In contrast, significantly larger ^19^F Δδ values (up to 18.1 ppm) are observed when G6P is substituted by a non-native hexose monophosphate (2-deoxy G6P or α-galactose 1-phosphate (αGal1P)), as the coordination of the MgF_3_^–^ moiety is substantially perturbed.^41^

**Figure 3:**
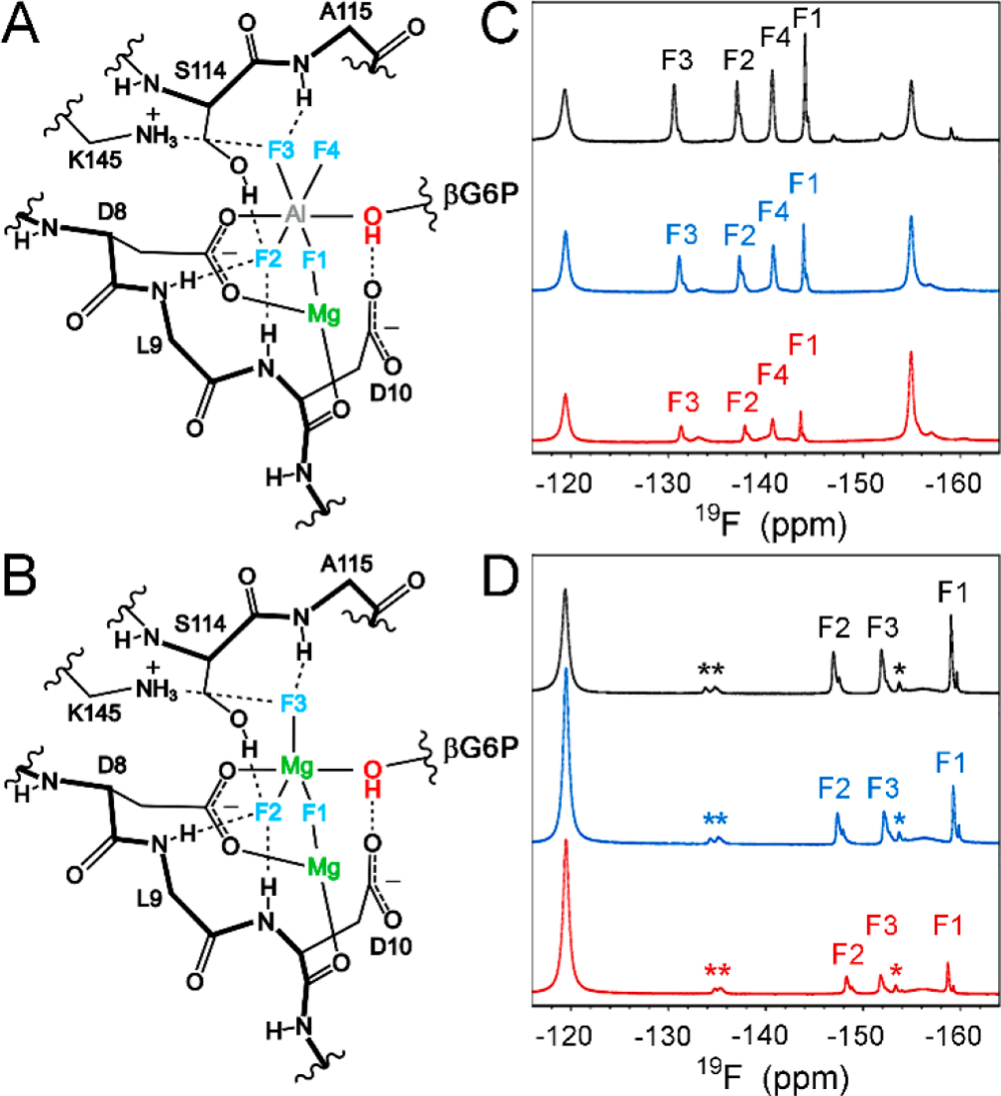
Active site coordination and ^19^F NMR spectra of the AlF_4_^–^ and MgF_3_^–^ moieties present in the βPGM:AlF_4_:G6P and βPGM:MgF_3_:G6P TSA complexes. (A,B) Schematic representation of (A) the square-planar AlF_4_^–^ moiety within the βPGM:AlF_4_:G6P TSA complexes and (B) the trigonal-planar MgF_3_^–^ moiety within the βPGM:MgF_3_:G6P TSA complexes, showing coordination by proximal site residues, the 1-hydroxyl group of βG6P, and Mg_cat_^2+^. Fluorine atoms have been labeled in accordance with IUPAC recommendations.^48^ (C,D) ^19^F NMR spectra for (C) βPGM_WT_:AlF_4_:G6P complex (black, top), βPGM_R49K_:AlF_4_:G6P complex (blue, middle), and βPGM_R49A_:AlF_4_:G6P complex (red, bottom) and (D) βPGM_WT_:MgF_3_:G6P complex (black, top), βPGM_R49K_:MgF_3_:G6P complex (blue, middle), and βPGM_R49A_:MgF_3_:G6P complex (red, bottom), acquired in standard NMR buffer containing 1 mM βPGM, 15 mM NaF, (3 mM AlCl_3_), and 20 mM G6P. Fluorine resonances corresponding to the AlF_4_^–^ and MgF_3_^–^ moieties have been labeled accordingly and are reported in Table 2. Small shoulders situated upfield (right) of the main resonances result from primary solvent induced isotope shifts arising from 10% v/v ^2^H_2_O present in the samples.^35^ Resonances indicated by asterisks correspond to an alternative conformation of the βPGM:MgF_3_:G6P TSA complexes.^32^ Free F^–^ resonates at −119 ppm, and free AlF_*x*_ species resonate at −155 ppm.

One-dimensional ^19^F NMR spectra of the βPGM_R49K_:AlF_4_:G6P and βPGM_R49A_:AlF_4_:G6P TSA complexes revealed four protein-bound ^19^F resonances, which were readily assigned according to their chemical shift ranges and their solvent induced isotope shifts (Figure 3A,C and Table 2).^35,37,40^ When compared with the βPGM_WT_:AlF_4_:G6P TSA complex, the observed Δδ values for the βPGM_R49K_:AlF_4_:G6P and βPGM_R49A_:AlF_4_:G6P TSA complexes showed a slight chemical shift change to a lower frequency for F2 (Δδ_R49K_ = −0.3 ppm and Δδ_R49A_ = −0.8 ppm) and F3 (Δδ_R49K_ = −0.5 ppm and Δδ_R49A_ = −0.7 ppm), a slight shift to a higher frequency for F1 (Δδ_R49K_ = +0.1 ppm and Δδ_R49A_ = +0.4 ppm), and no change for F4 (Figure 3A,C and Table 2). Equivalent ^19^F NMR spectra for the βPGM_R49K_:MgF_3_:G6P and βPGM_R49A_MgF_3_:G6P TSA complexes showed three protein-bound ^19^F resonances that were readily assigned using the βPGM_WT_:MgF_3_:G6P TSA complex (Figure 3B,D and Table 2).^35,37,40^ Comparisons of ^19^F frequencies revealed a similar shift to a lower frequency for F2 (Δδ_R49K_ = −0.4 ppm and Δδ_R49A_ = −1.3 ppm), whereas F3 (Δδ_R49K_ = −0.2 ppm and Δδ_R49A_ = +0.1 ppm) and F1 (Δδ_R49K_ = −0.2 ppm and Δδ_R49A_ = +0.3 ppm) showed small Δδ values with opposite shielding effects. Furthermore, at identical βPGM and G6P concentrations, the differences observed in ^19^F peak intensities between all of the TSA complexes (Figure 3C,D) mirror the reduction in binding affinity reported by the apparent *K*_d_ (G6P) values (Table 1 and Figure S5). Significantly, all of the observed |Δδ| values are small (<1.7 ppm), and it is likely that these result from subtle modifications in the chemical environment of the fluorine nuclei (Figure 2 and Figure 3A,B) due to small differences in the positioning of G6P and proximal site residues when the coordination of the phosphodianion group in the distal site is perturbed.

**Table 2:** ^19^F Chemical Shifts (ppm) Observed for the AlF_4_^–^ and MgF_3_^–^ Moieties Present in the βPGM:AlF_4_:G6P and βPGM:MgF_3_:G6P TSA Complexes

TSA complex	F1	F2	F3	F4
βPGM_WT_:AlF_4_:G6P	–144.0	–137.0	–130.6	–140.7
βPGM_R49K_:AlF_4_:G6P	–143.9	–137.3	–131.1	–140.8
βPGM_R49A_:AlF_4_:G6P	–143.6	–137.8	–131.3	–140.7
βPGM_WT_:MgF_3_:G6P	–159.0	–147.0	–151.9	
βPGM_R49K_:MgF_3_:G6P	–159.2	–147.4	–152.1	
βPGM_R49A_:MgF_3_:G6P	–158.7	–148.3	–151.8	

**Figure 4:**
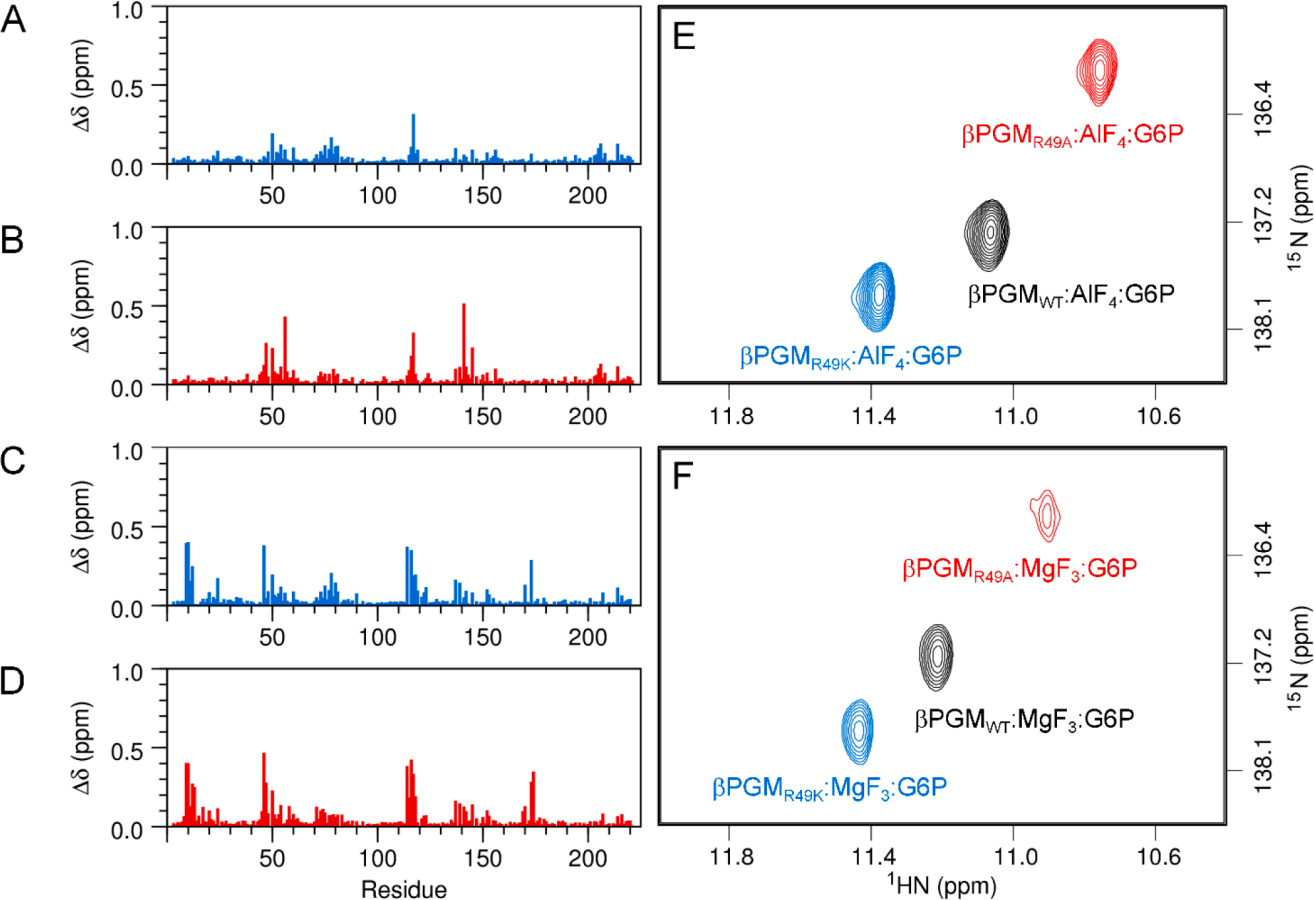
Chemical shift perturbations arising from R49 side chain substitution in the βPGM:AlF_4_:G6P and βPGM:MgF_3_:G6P TSA complexes. (A–D) Weighted chemical shift changes of the backbone amide group are calculated for each residue as Δδ = [(δ_HN–X_ – δ_HN–Y_)^2^ + (0.13 × (δ_N–X_ – δ_N–Y_))^2^]^1/2^, where X and Y are the two complexes being compared. (A) Δδ values between βPGM_R49K_:AlF_4_:G6P and βPGM_WT_:AlF_4_:G6P complexes. (B) Δδ values between βPGM_R49A_:AlF_4_:G6P and βPGM_WT_:AlF_4_:G6P complexes. (C) Δδ values between βPGM_R49K_:MgF_3_:G6P and βPGM_WT_:MgF_3_:G6P complexes. (D) Δδ values between βPGM_R49A_:MgF_3_:G6P and βPGM_WT_:MgF_3_:G6P complexes. The small magnitude (0.1–0.5 ppm) of the Δδ values indicates that the extent of perturbation across the active site upon R49 side chain substitution in a fully closed, near-transition state conformation is not substantial. (E,F) Overlays of a section of ^1^H^15^N-TROSY NMR spectra for the βPGM:AlF_4_:G6P and βPGM:MgF_3_:G6P TSA complexes highlighting the behavior of residue K117. (E) βPGM_WT_:AlF_4_:G6P complex (black), βPGM_R49K_:AlF_4_:G6P complex (blue), and βPGM_R49A_:AlF_4_:G6P complex (red). (F) βPGM_WT_:MgF_3_:G6P complex (black), βPGM_R49K_:MgF_3_:G6P complex (blue), and βPGM_R49A_:MgF_3_:G6P complex (red). The backbone amide group of residue K117 coordinates one of the phosphodianion oxygen atoms of G6P. In the βPGM_R49K_:AlF_4_:G6P and βPGM_R49K_:MgF_3_:G6P TSA complexes, the K117 peak is further shifted to higher ^1^H and ^15^N frequencies consistent with a slight shortening of this hydrogen bond due to small changes in the position of the phosphodianion group upon R49 side chain substitution. In the βPGM_R49A_:AlF_4_:G6P and βPGM_R49A_:MgF_3_:G6P TSA complexes, this peak is shifted in the opposite direction to lower frequencies, in accord with the slight lengthening of this hydrogen bond.

Additionally, the chemical shift assignments for the backbone amide groups were determined for the βPGM_R49K_:AlF_4_:G6P, βPGM_R49A_:AlF_4_:G6P, βPGM_R49K_:MgF_3_:G6P, and βPGM_R49A_:MgF_3_:G6P TSA complexes by comparison with their βPGM_WT_ TSA counterparts. Weighted chemical shift changes relative to the βPGM_WT_:AlF_4_:G6P and βPGM_WT_:MgF_3_:G6P TSA complexes are localized to discrete protein regions across the four comparisons (Figure 4A–D and Figure S6 A–D). Residues that comprise the two interdomain hinges (D15–T16 and V87–S88) show only small Δδ values (0.1–0.2 ppm), indicating that the degree of domain closure is consistent. The substrate specificity loop (K45–S52)^49^ and a cap domain α-helix (A73–N78) show Δδ values arising from R49 side chain substitution, which mirror the magnitude of those observed for substrate-free βPGM_R49K_ and substrate-free βPGM_R49A_ (Figure S1). In the fully closed TSA complexes, a small propagation of the effect (0.1–0.3 ppm) of R49 side chain substitution is reflected in the D137–P148 loop due to the close proximity of the cap and core domains, and small Δδ values (0.1–0.5 ppm) are observed in the S114–N118 loop interconnecting the proximal and distal sites (Figures 2, 4A–D, and S6A–D). Residues S114 and A115 coordinate the AlF_4_^–^ and MgF_3_^–^ moieties. Residue S116 forms key hydrogen bond interactions with both S114 and one of the phosphodianion oxygen atoms of G6P, and additional coordination of the phosphodianion group in the distal site is mediated by residues K117 and N118 (Figure 2 and Movie S1). In particular, the local effects of differential coordination of the phosphodianion group upon R49 side chain substitution is evident through the behavior of the backbone amide group of K117, owing to its hydrogen bond with one of the phosphodianion oxygen atoms of G6P (Figures 2 and 4E,F and Movie S1). In the βPGM:MgF_3_:G6P TSA complexes, there is some further propagation through the MgF_3_^–^ moiety to the backbone amide groups of L9 and D10, together with residues coordinating Mg_cat_^2+^ (Figures 2, 4C,D, and S6C,D). However, taken together, the small magnitude of the ^1^H, ^15^N, and ^19^F chemical shifts changes indicates that the extent of perturbation across the active site upon R49 side chain substitution in the TSA complexes is not substantial, and therefore βPGM_R49K_ and βPGM_R49A_ can adopt fully closed, near-transition state conformations in solution.

### Catalytic Activity of βPGM_R49K_ and βPGM_R49A_

**Figure 5:**
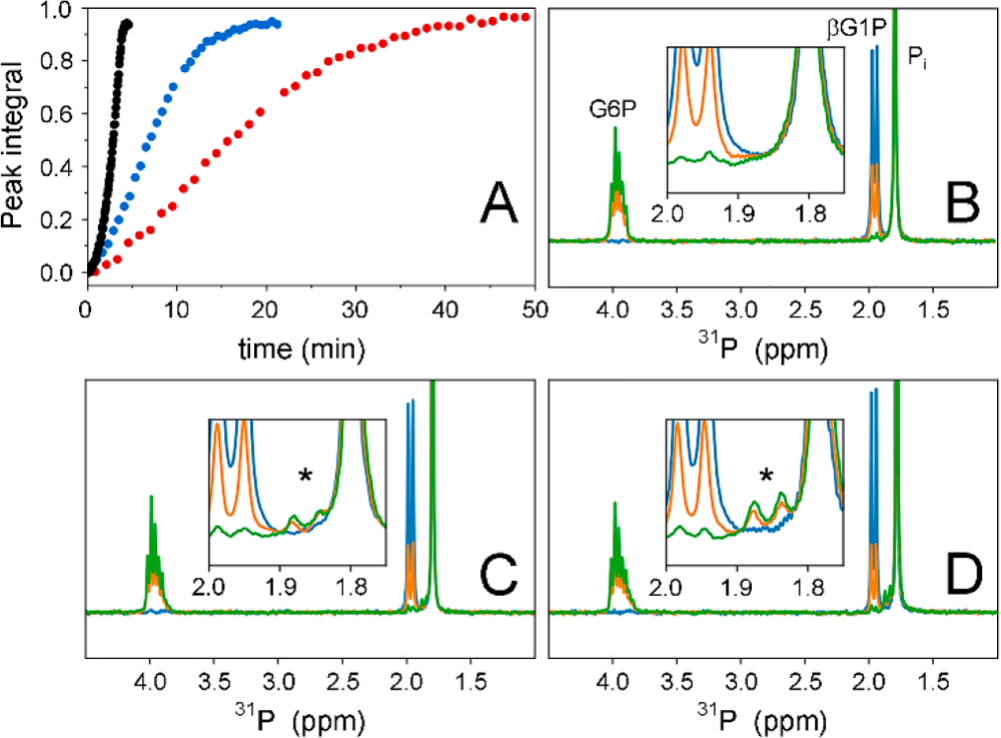
Catalytic activity of βPGM_WT_, βPGM_R49K_, and βPGM_R49A_ monitored using ^31^P NMR spectra. (A) Reaction kinetics for the equilibration of saturating 10 mM βG1P with G6P in standard kinetic buffer catalyzed by 0.05 μM βPGM_WT_ (black circles, left), 0.5 μM βPGM_R49K_ (blue circles, middle), or 1.0 μM βPGM_R49A_ (red circles, right). The reaction was initiated by 20 mM AcP and timed immediately after its addition. Normalized integral values for the G6P peak are plotted as a function of time. To facilitate comparison between the kinetic profiles, the time axes for βPGM_WT_ and βPGM_R49K_ are scaled by the βPGM_WT_/βPGM_R49A_ and βPGM_R49K_/βPGM_R49A_ concentration ratios, respectively. (B–D) Overlays of ^31^P NMR spectra from the beginning (blue), midpoint (orange), and end (green) of the kinetic profiles for (B) βPGM_WT_, (C) βPGM_R49K_, and (D) βPGM_R49A_. Corresponding changes in βG1P and G6P peak intensities are observed as the reactions progress. Inlays highlight the formation of up to ∼1 mM βG16BP reaction intermediate (black asterisks, 1-phosphate doublet of βG16BP) during the course of the reactions catalyzed by βPGM_R49K_ and βPGM_R49A_, whereas for the βPGM_WT_ reaction, βG16BP accumulation is not observed.

The consequences of R49 side chain substitution on enzyme catalytic activity were investigated using kinetic assays. ^31^P NMR time-course experiments were used to monitor the production of G6P by βPGM_R49K_ and βPGM_R49A_ in the presence of a saturating concentration of βG1P substrate (10 mM; Figure 1A). In vitro, 20 mM acetyl phosphate (AcP) is required as a phosphorylating agent to initiate the reaction, as the half-life of βPGM^P^ is ∼30 s (Figure S7).^34^ The ^31^P NMR peak integrals for G6P were normalized and plotted as a function of time. The resulting kinetic profiles were similar in shape to that for the βPGM_WT_ time course (Figure 5A). Subsequent fitting of their steady-state linear segments yielded observed catalytic rate constants for βPGM_R49K_ (*k*_obs_ = 14.8 ± 1 s^–1^) and βPGM_R49A_ (*k*_obs_ = 5.9 ± 0.5 s^–1^). These *k*_obs_ values represent a 5-fold and 12-fold reduction compared to that for βPGM_WT_ (*k*_obs_ = 70 ± 1 s^–1^) measured under the same conditions (Table 1). The trend in the reduced *k*_obs_ values for βPGM_R49K_ and βPGM_R49A_ is consistent with the increases in the apparent *K*_d_ (G6P) values for the βPGM_R49K_:AlF_4_:G6P and βPGM_R49A_:AlF_4_:G6P TSA complexes, implying that enzyme catalytic activity is partially affected by differential coordination of the phosphodianion group in the distal site.

A previously reported kinetic characterization of βPGM_WT_ catalytic activity identified the presence of a lag phase prior to the attainment of steady-state kinetic behavior,^34^ caused by two independent kinetic components. The first component is an allomorphic effect (arising from *cis–trans* proline isomerization at the K145–P146 peptide bond) operating over a short time frame (<5 min), where the full rate of catalysis is delayed until the concentration of the βG16BP intermediate is sufficiently elevated to phosphorylate βPGM_WT_ efficiently.^28^ The second component is due to substrate inhibition and operates over a longer time frame (5–15 min), where βG1P associates with substrate-free βPGM_WT_ (*K*_i_ (βG1P) = 1510 ± 100 μM) forming an inhibited complex (Figure S7).^28^ For βPGM_R49K_ and βPGM_R49A_, the allomorphic component of the lag phase persists in the early parts of the kinetic profiles, while differences in the βG1P inhibition component are more difficult to distinguish as the *k*_obs_ values are smaller. Furthermore, the concentration requirements of the ^31^P NMR experimental setup precluded the use of a range of βG1P concentrations to deconvolute *k*_obs_ into *k*_cat_ and *K*_i_ (βG1P). Surprisingly, the ^31^P NMR spectra acquired to monitor βPGM_R49K_ and βPGM_R49A_ catalysis show the presence of the βG16BP intermediate building to measurable concentrations in the reaction sample, whereas equivalent experiments recorded using βPGM_WT_ indicate that the steady-state concentration of βG16BP is too low to be detected because of its rapid conversion to G6P (Figure 5B,C,D). These observations demonstrate that binding of the βG16BP intermediate is also compromised by R49 side chain substitution in the distal site.

Further kinetic experiments were conducted for βPGM_R49K_ and βPGM_R49A_ to investigate the dependence of the steady-state reaction velocity on βG1P concentration. Here, a glucose 6-phosphate dehydrogenase coupled assay was used to monitor the conversion of βG1P to G6P with AcP present as the phosphorylating agent (Figure S7).^38^ βG16BP^28,50^ could not be used as a phosphorylating agent since its affinity is substantially weakened and concentrations greater than 10 μM result in multimeric interactions with Mg^2+^ ions present in the buffer.^28,38,50^ As for βPGM_WT_, the kinetic profiles for βPGM_R49K_ and βPGM_R49A_ display an initial allomorphic lag phase,^28^ whereas the βG1P inhibition component acting over longer timeframes prior to steady-state kinetics is much less prominent than for βPGM_WT_ (Figure S8A,B,C). Unfortunately, the weak βG1P affinity of both βPGM_R49K_ and βPGM_R49A_ prevented the determination of reliable kinetic parameters over the experimentally accessible βG1P concentration range (Figure S8D,E,F). However, a linear fit to the initial data points of each Michaelis–Menten plot allowed the *k*_cat_/*K*_m_ ratio to be derived for βPGM_WT_ (*k*_cat_/*K*_m_ = 0.29 s^–1^·μM^–1^), βPGM_R49K_ (*k*_cat_/*K*_m_ = 0.05 s^–1^·μM^–1^), and βPGM_R49A_ (*k*_cat_/*K*_m_ = 0.02 s^–1^·μM^–1^; Table 1 and Figure S8D,E,F). These *k*_cat_/*K*_m_ ratios represent a 6-fold and 15-fold reduction compared to that for βPGM_WT_ under the same conditions, which mirrors the reduction in *k*_obs_ values determined using ^31^P NMR time-course experiments. In conclusion, the kinetics results obtained from the ^31^P NMR time-course experiments and the coupled assays indicate that R49 side chain substitution in the distal site mainly impairs binding of βG16BP and βG1P, rather than reducing catalytic activity. Furthermore, such perturbation also alleviates βG1P inhibition.

### βPGM_D170N_ Binds βG1P in a Fully Closed Inhibited Complex

**Figure 6:**
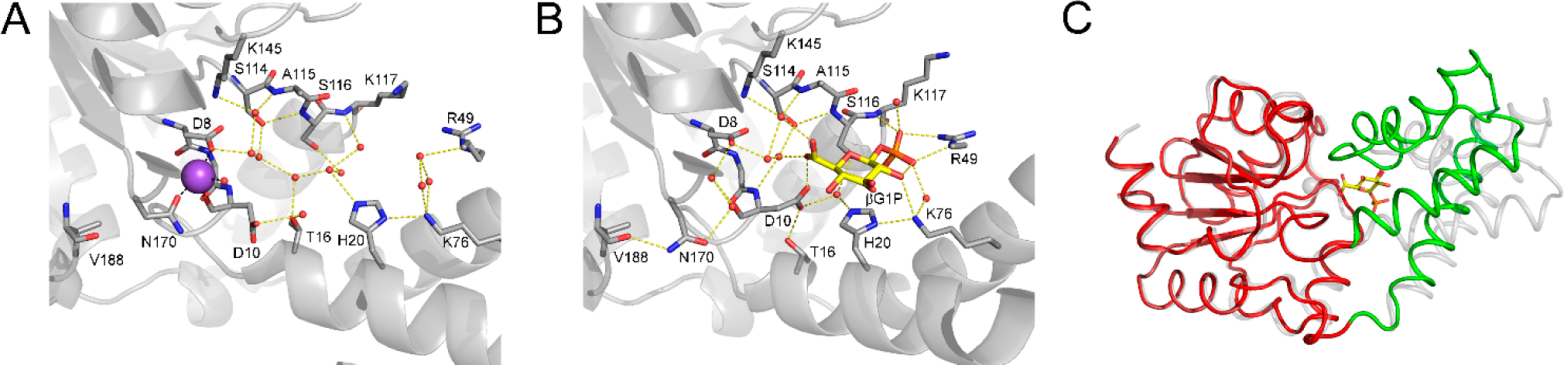
Crystal structure comparisons of substrate-free βPGM_D170N_ and the inhibited βPGM_D170N_:βG1P complex. (A) Active site details of substrate-free βPGM_D170N_ (PDB 6HDF), with selected residues (sticks) and structural waters (red spheres) shown, and a Na^+^ atom (purple sphere) occupying the Mg_cat_^2+^ site. (B) Active site details of the inhibited βPGM_D170N_:βG1P complex (PDB 6HDG), with selected residues (sticks), structural waters (red spheres), and βG1P (gold carbon atoms) illustrated. The 6-hydroxyl group of βG1P in the proximal site has two arrangements resolved for the C5–C6 bond. Yellow dashes indicate hydrogen bonds and black dashes show metal ion coordination. For R49, the Cα and Cβ atoms have been omitted for clarity. The side chain of residue N118, which coordinates one of the phosphodianion oxygen atoms of βG1P, has also been omitted for clarity. (C) Superposition of substrate-free βPGM_D170N_ (PDB 6HDF) and the inhibited βPGM_D170N_:βG1P complex (PDB 6HDG) on the core domain showing the extent of domain closure. The protein backbone of substrate-free βPGM_D170N_ is displayed as a pale gray ribbon. The protein backbone of the inhibited βPGM_D170N_:βG1P complex is depicted as a ribbon, with the core (red) and cap (green) domains indicated, and βG1P shown as sticks (gold carbon atoms).

To investigate the role of the distal site in the formation of the inhibited βPGM:βG1P complex, crystallization trials were attempted. Since βG1P readily equilibrates with G6P in solution in the presence of βPGM_WT_, the nonhydrolyzable β-glucose 1-fluorophosphonate mimic was used in cocrystallization experiments,^37^ but all trials were unsuccessful. Therefore, the partially inactivated D170N variant (βPGM_D170N_)^50^ was used, where perturbation of the Mg_cat_^2+^ site was achieved through an anionic to neutral side chain substitution (Figure 2). A comparison of ^1^H^15^N-TROSY NMR spectra indicated that substrate-free βPGM_D170N_ has similar solution properties and overall protein fold to substrate-free βPGM_WT_, including the slow-exchange behavior that arises from *cis–trans* proline isomerization at the K145–P146 peptide bond.^50^ Substrate-free βPGM_D170N_ was crystallized (1.4 Å resolution, PDB 6HDF, Table S1), and its structure shows an open domain arrangement that closely resembles other substrate-free βPGM structures (Figures 6A, S2, and S3). However, in both monomers of the asymmetric unit, a Na^+^ ion is present instead of Mg_cat_^2+^ in the proximal site. βPGM_D170N_ showed significantly reduced catalytic activity (*k*_obs_ = 3.0 × 10^–3^ s^–1^), a decrease in Mg_cat_^2+^ affinity (apparent *K*_m_ (Mg^2+^) = 690 ± 110 μM), together with an increase in βG1P affinity (apparent *K*_m_ (βG1P) = 6.9 ± 1.0 μM), and a similar level of βG1P inhibition (apparent *K*_i_ (βG1P) = 1540 ± 170 μM)^50^ compared to the values obtained for βPGM_WT_ under similar conditions (*k*_cat_ = 382 ± 12 s^–1^, *K*_m_ (Mg^2+^) = 180 ± 40 μM, *K*_m_ (βG1P) = 91 ± 4 μM, and *K*_i_ (βG1P) = 1510 ± 100 μM).^28^ These kinetic parameters indicate that the side chain substitution in βPGM_D170N_ primarily perturbs Mg_cat_^2+^ binding in the proximal site, resulting in a reduction in catalytic activity. However, binding of βG1P, both during the catalytic cycle and in the formation of the inhibited complex, is only modestly affected. Overall, therefore, βPGM_D170N_ appears to be a suitable candidate with which to study the inhibited βPGM:βG1P complex.

Crystallization trials involving βPGM_D170N_ along with MgF_3_^–^ and G6P were prepared to obtain a structure of the βPGM_D170N_:MgF_3_:G6P TSA complex. The resulting structure, however, was a βPGM_D170N_:βG1P complex (1.2 Å resolution, PDB 6HDG, Table S1, Figures 6B, S2, S3, and S9A). The presence of βG1P in the crystallization buffer is a result of βPGM_D170N_ reversible catalytic activity,^50^ which is a process that has been reported previously for βPGM_WT_ in crystallization experiments.^36,51^ The trigonal-planar MgF_3_^–^ moiety mimicking the transferring phosphoryl group in the proximal site was absent. Inspection of the electron density map indicates that neither a Mg^2+^ ion nor a Na^+^ ion is coordinated in the Mg_cat_^2+^ site, despite the inclusion of 5 mM Mg^2+^ and ∼200 mM Na^+^ ions in the crystallization buffer. Instead, the side chain of N170 is rotated 103° about χ_1_ such that the carboxamide group forms a hydrogen bond with the backbone carbonyl group of V188, rather than coordinating a cation in the Mg_cat_^2+^ site, as observed for the side chain of D170 in βPGM_WT_ (Figures 6B and S9A). The phosphodianion group of βG1P is coordinated in the distal site by the backbone amide group of K117, the side chain hydroxyl group of S116, the side chain carboxamide group of N118, and the guanidinium group of R49, in an analogous arrangement to that present in the βPGM_WT_:MgF_3_:βG1phosphonate TSA complex (PDB 4C4R).^37^ Also, a comparable extensive hydrogen bond network involving residues of the active site coordinates three hexose ring hydroxyl groups of βG1P directly, rather than being mediated by water molecules as observed in equivalent βPGM:MgF_3_:G6P TSA complexes.^37^ In the proximal site, the 6-hydroxyl group of βG1P has two arrangements resolved for the C5–C6 bond, which differ in their rotation by ∼140°. This arrangement facilitates hydrogen bonding separately with two of the three water molecules that now occupy the location of the missing trigonal-planar MgF_3_^–^ moiety (Figure 6B and Figure S9A). Furthermore, such proximity of the C6–O6 bond of βG1P to the site of phosphoryl transfer allows alignment with the Oδ1 carboxylate atom of residue D8 (nucleophile) and engagement of residue D10 (general acid–base) in the active site, along with coordination of residue T16 in a manner associated with full domain closure.^38^ Therefore, this structure represents a ground state complex with a fully closed, near-transition state conformation (Figures 6C, S2, and S3), which serves as an excellent model for the inhibited βPGM_WT_:βG1P complex. The population of such a stable complex is consistent with the βG1P inhibition component of the lag phase observed in kinetic experiments.

### βPGM_WT_ Coordinates a Phosphate Anion in the Distal Site

A βPGM_WT_:P_i_ complex was obtained using 10 mM sodium phosphate in the crystallization buffer, and its structure was determined (1.8 Å resolution, PDB 6H93, Table S1, Figures S2 and S3). The two monomers in the asymmetric unit both display an open conformation, together with a phosphate anion coordinated in the distal site by the guanidinium group of R49 and the alkylammonium side chains of K76 (via a water molecule) and K117 (Figure S9C,D). These residues occupy identical locations to those present in substrate-free βPGM, and their Cα atom positions are *ca*. 3 Å more separated than their equivalent positions in the fully closed TSA complexes. Moreover, there was no evidence of a phosphate anion coordinated in the proximal site. Hence, the open βPGM_WT_:P_i_ complex presents an initial mode for phosphodianion group interaction in the distal site, which is independent of a covalently attached hexose group, and it offers a plausible mechanism for the phosphate anion inhibition of βPGM_WT_ catalytic activity reported previously.^38^ Furthermore, the open βPGM_WT_:P_i_ complex indicates that binding of a phosphate anion in isolation cannot facilitate the transition to a fully closed complex.

## Discussion

Side chain substitution of the guanidinium group of R49 in either βPGM_R49K_ or βPGM_R49A_ impairs G6P, βG1P, and βG16BP binding and leads to the partial alleviation of βG1P inhibition. However, these changes result in only modest reductions in the *k*_obs_ values compared to that of βPGM_WT_. While these substitutions induce an alternative coordination of the phosphodianion group in the distal site via the recruitment of neighboring alkylammonium side chains in the TSA complexes involving G6P, the proximal site architecture, expulsion of water from the active site, and degree of domain closure are equivalent to βPGM_WT_ TSA complexes. Hence, despite R49 side chain substitution, the coordination of the phosphodianion group in the distal site is sufficient to allow a fully closed, near-transition state conformation.

In the phosphodianion-driven enzyme-activation framework,^21−24^ the energy derived from a cation–phosphodianion interaction in a distal site is used to stabilize the closed active form. An underlying assumption of this framework is that once E_C_:S has been achieved, the organization of catalytic groups within the desolvated active site is sufficient for catalysis to occur, implying that the intrinsic binding energy of the phosphodianion group in the distal site does not also specifically reduce the transition state energy barrier for the chemical step.^24^ Hence, the only consequence of cationic side chain substitution is destabilization of E_C_:S. Experimental evidence to support such an assumption is observed in βPGM through only modest Δδ values for the ^19^F resonances of the AlF_4_^–^ and MgF_3_^–^ moieties in the βPGM_R49K_ and βPGM_R49A_ TSA complexes compared to their βPGM_WT_ counterparts. Additionally, the small Δδ values of the observed backbone amide resonances are not consistent with inherent difficulties in the adoption of a fully closed, near-transition state conformation. Therefore in βPGM, the intrinsic binding energy of the phosphodianion group is utilized overwhelmingly to stabilize E_C_:S, rather than to specifically stabilize the transition state of the chemical step. Moreover, these results indicate that any intersite communication within the active site to promote catalysis is not substantial.

The small extent of intersite communication through the near-transition state structure enables the kinetic consequences of distal site perturbations to be separated from those elicited by proximal site perturbations. In substrate-free βPGM_D170N_, both Mg_cat_^2+^ binding and catalytic activity are impaired, while *K*_m_ (βG1P) and *K*_i_ (βG1P) are only modestly affected.^50^ Structurally, the inhibited βPGM_D170N_:βG1P complex adopts a fully closed, near-transition state conformation. This observation is consistent with the βG1P-dependent lag phase operating in βPGM_WT_,^28,34,38^ which is partially alleviated in kinetic assays involving βPGM_R49K_ and βPGM_R49A_. Additionally, αGal1P is another hexose 1-phosphate that behaves as a competitive inhibitor of βPGM_WT_ (*K*_i_ (αGal1P) = 30 μM).^52^ Although αGal1P is a poor surrogate for βG1P, owing to differences in stereochemistry at both the C1 and C4 positions, the βPGM_WT_:αGal1P complex can adopt a similar fully closed, near-transition state conformation (PDB 1Z4O and PDB 1Z4N, Figure S9B).^52^ Therefore, coordination of the hexose 1-phosphate phosphodianion group in the distal site leads to domain closure, whereas a free phosphate anion does not stabilize a fully closed complex. Additionally, the observation of a βPGM_WT_:P_i_ complex suggests that the residue side chains comprising the distal site are preorganized to provide the initial mode of phosphodianion group interaction (Figure S9C,D). In summary, binding of the phosphodianion group of the substrates, the reaction intermediate, or non-native hexose monophosphates in the distal site facilitates the transition to a fully closed, near-transition state conformation, but at the expense of introducing hexose 1-phosphate inhibition.

**Figure 7:**
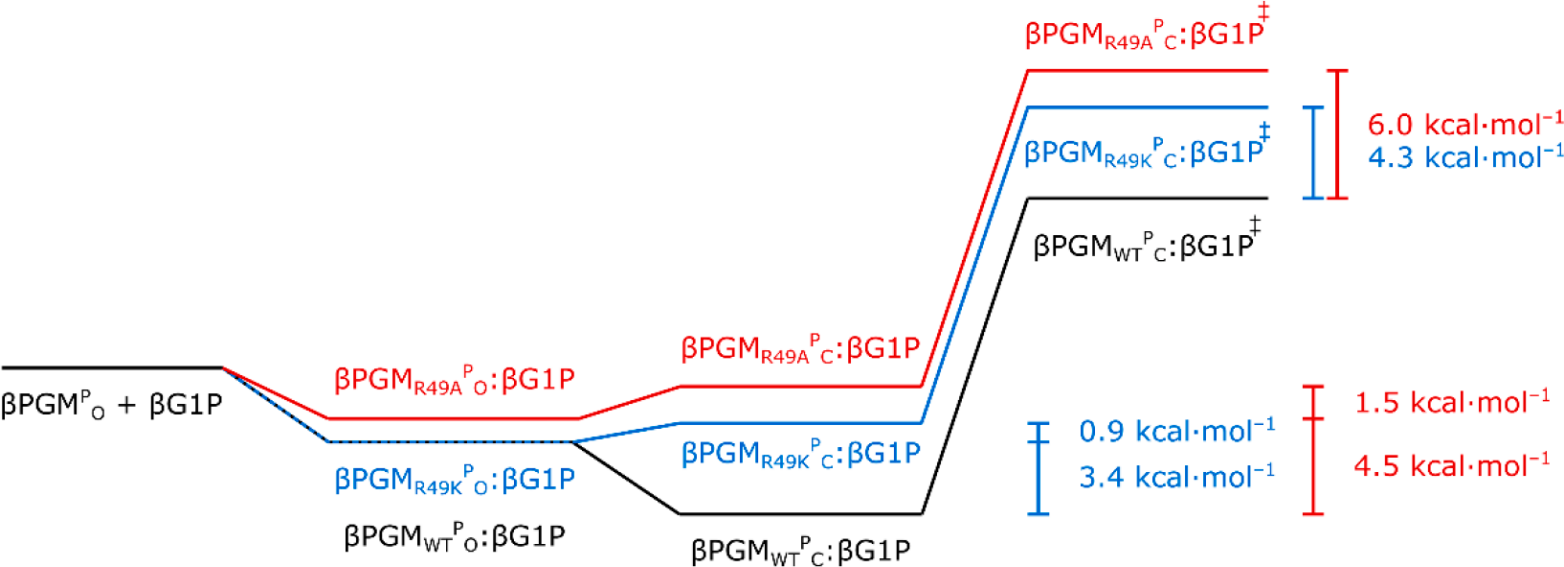
Free energy reaction profiles for βPGM illustrating the effect of R49 side chain substitution on the kinetic parameters. The apparent *K*_d_ (G6P) and *k*_obs_ values were used to estimate the cation–phosphodianion interaction energy and its role in the stabilization of the transition state (Table 1). βPGM^P^_O_ corresponds to the open phospho-enzyme, the βPGM^P^_O_:βG1P complex corresponds to the open inactive form, the βPGM^P^_C_:βG1P complex corresponds to the closed active form, and the βPGM^P^_C_:βG1P^‡^ complex corresponds to the transition state of phosphoryl transfer. The energy of the βPGM_WT_^P^_O_:βG1P complex is estimated to be similar to that of the βPGM_R49K_^P^_O_:βG1P complex, since both retain a cationic charge in the distal site.

The free energy contribution of the cation-phosphodianion interaction in the distal site (ΔΔ*G*) to the stabilization of E_C_:S can be estimated by measuring the change in the stability of both the Michaelis complex (ΔΔ*G*_S_) and the transition state (ΔΔ*G*^‡^) on perturbation of the key cationic side chain. This analysis relies on the assumption that the energy contributions of individual residues are approximately additive and their interactions with the substrate are not significantly cooperative.^53^ When comparing the ΔΔ*G*_S_ and ΔΔ*G*^‡^ components of the cation–phosphodianion interaction energy, one of two scenarios are observed that reveal the impact of the perturbation on the catalytic cycle: (1) a dominant ΔΔ*G*_S_ component indicates that E_C_:S becomes destabilized and the identity of the Michaelis complex switches from E_C_:S to E_O_:S, with E_O_:S → E_C_:S becoming part of the rate-limiting step of the reaction, or (2) a dominant ΔΔ*G*^‡^ component indicates that E_O_:S remains as the Michaelis complex.^18−20^ In βPGM, the small extent of intersite communication observed in the near-transition state conformations implies that the roles of the phosphodianion group binding residues in the distal site and the catalytic residues in the proximal site are largely independent. Consequently, the apparent *K*_d_ (G6P) and *k*_obs_ values are used to estimate the ΔΔ*G*_S_ and ΔΔ*G*^‡^ components of the impact on stabilization of E_C_:S following R49 side chain substitution (Table 1). For both βPGM_R49K_ and βPGM_R49A_, the ΔΔ*G* values derived using the kinetic parameters are partitioned into a larger ΔΔ*G*_S_ component and a smaller ΔΔ*G*^‡^ component (Table 1 and Figure 7). Such a partitioning implies that the Michaelis complex of βPGM_WT_ is E_C_:S but switches to E_O_:S in βPGM_R49K_ and βPGM_R49A_. However, the recruitment of the side chain of K117 in coordinating the phosphodianion group in the distal site observed in the βPGM_R49A_:AlF_4_:G6P TSA complex provides redundancy in stabilizing E_C_:S. As a consequence, the actual ΔΔ*G* value for βPGM_R49A_ is larger than measured.

The dominant ΔΔ*G*_S_ component implies that the energy derived from binding the phosphodianion group of βG1P in the distal site of the open phospho-enzyme (βPGM^P^_O_) is utilized primarily to facilitate a shift in the equilibrium from an open inactive βPGM^P^_O_:βG1P complex to a closed active βPGM^P^_C_:βG1P complex (Figure 7). The energy difference between the βPGM^P^_C_:βG1P complex and the βPGM^P^_C_:βG1P^‡^ transition state is not significantly affected by each of the R49 side chain substitutions, as demonstrated by the minimal extent of perturbation across the active site in near-transition state complexes. The change in stability of the respective βPGM^P^_C_:βG1P^‡^ transition states (Table 1 and Figure 7) results from the differential stability of the corresponding closed βPGM^P^_C_:βG1P complexes.

Structural evidence of the adoption of E_C_:S for βPGM^P^ upon binding either βG1P or G6P is provided through comparisons between the open βPGM_WT_:BeF_3_ complex (a mimic of βPGM^P^, PDB 2WFA) and either the closed βPGM_WT_:BeF_3_:βG1P (PDB 2WF8) or the closed βPGM_WT_:BeF_3_:G6P (PDB 2WF9) complexes (Figure S2).^36^ Likewise, the adoption of E_C_:S for substrate-free βPGM upon binding βG16BP in either orientation is illustrated by comparisons between substrate-free βPGM_WT_ (PDB 2WHE)^35^ and either of the closed βPGM_D10N_:βG16BP complexes (PDB 5OK0 and PDB 5OK1; Figure S2).^38^

**Figure 8:**
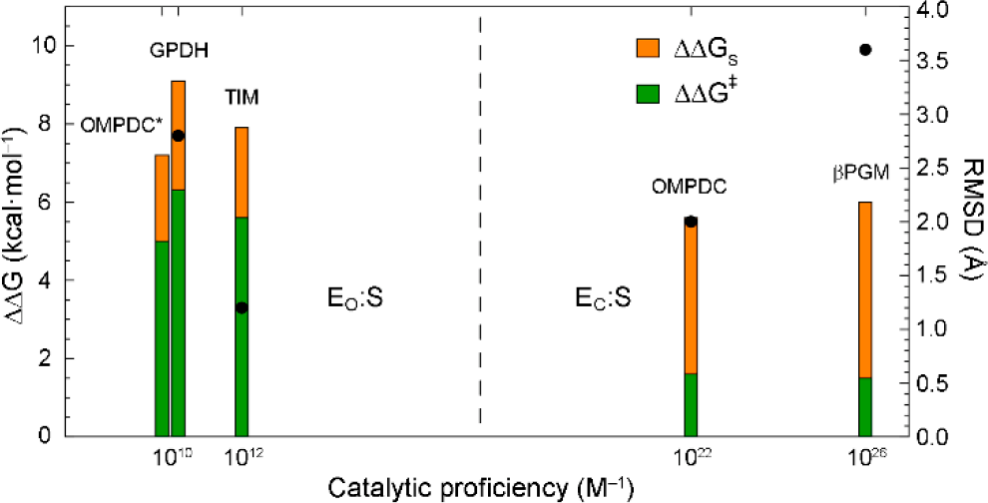
Relationship between the partitioning of the cation–phosphodianion interaction energy, the magnitude of the conformational change upon adoption of E_C_:S, and the catalytic proficiency of OMPDC*, GPDH, TIM, OMPDC, and βPGM. The free energy contribution of the cation–phosphodianion interaction in the distal site to the adoption of E_C_:S was estimated by measuring the change in the stability of both the Michaelis complex (ΔΔ*G*_S_; orange bars) and the transition state (ΔΔ*G*^‡^; green bars) on substitution of the key cationic side chain (Table 3). The identity of the Michaelis complex is indicated. The extent of the conformational change upon adoption of E_C_:S is reported as pairwise non-H RMSD values derived from the structures of open and closed enzymes (black circles). For GPDH, RMSD = 2.8 Å (PDB 6E8Z chain A and PDB 6E90 chain A).^25^ For TIM, RMSD = 1.3 Å (PDB 3TIM chain A and PDB 1IIH chain B).^21^ For OMPDC, RMSD = 2.0 Å (PDB 1DQW and PDB 1DQX).^26^ For βPGM, RMSD = 3.6 Å (PDB 2WFA and PDB 2WF8).^36^ The catalytic proficiency is calculated either as (*k*_ex_/*K*_d_)/*k*_non_, (*k*_cat_/*K*_m_)/*k*_non_, or (*k*_obs_/*K*_d_)/*k*_non_, where *k*_non_ is the rate constant for the corresponding spontaneous noncatalyzed reaction (Table 3).

The dominant ΔΔ*G*_S_ component in βPGM mirrors that reported previously for OMPDC (Table 3).^19^ In contrast, both GPDH and TIM display dominant ΔΔ*G*^‡^ components (Table 3).^18,20^ Hence, for both βPGM and OMPDC, the Michaelis complex is E_C_:S and, for both GPDH and TIM, the Michaelis complex is E_O_:S. Furthermore, the identity of the Michaelis complex does not correlate with the complexity of the conformational change required upon adoption of E_C_:S, since βPGM, OMPDC, and GPDH all display large non-H atom RMSD values (>2.0 Å) between open and closed enzyme forms (Figures 6C, 8, and S2). Notably, OMPDC displays a dominant ΔΔ*G*^‡^ component for the proton–deuterium exchange reaction involving the non-native substrate 5-fluorouridine 5′-monophosphate, implying that for this reaction (OMPDC*; Table 3 and Figure 8), the Michaelis complex is E_O_:S.^19^ One distinguishing feature between OMPDC and OMPDC* is the substantial difference in their catalytic proficiencies. OMPDC*, GPDH, and TIM have catalytic proficiencies ranging between 10^10^ and 10^12^ M^–1^ (Table 3).^18,54−56^ In contrast, OMPDC and βPGM have catalytic proficiencies greater than 10^22^ M^–1^ (Table 3).^19,27,57,58^ Therefore, the identity of the Michaelis complex instead correlates with the catalytic proficiency of the enzyme (Figure 8). In conclusion, the analysis described here for βPGM, together with the data for GPDH, TIM, and OMPDC, supports a trend, whereby enzymes with high catalytic proficiencies involving phosphorylated substrates primarily utilize the cation–phosphodianion interaction energy for stabilization of E_C_:S.

**Table 3:** Cationic Side Chain Contribution to the Intrinsic Binding Energy of the Phosphodianion Group, Partitioned into ΔΔ*G*_S_ (kcal·mol^–1^) and ΔΔ*G*^‡^ Components (kcal·mol^–1^), Together with the Catalytic Proficiency of the Enzyme (M^–1^)

enzyme	ΔΔ*G*_S_	ΔΔ*G*^‡^	catalytic proficiency^a^
OMPDC*^b^	2.2	5.0	3 × 10^10^
GPDH^c^	2.8	6.3	7 × 10^10^
TIM^d^	2.3	5.6	2 × 10^12^
OMPDC^e^	4.0	1.6	4 × 10^22^
βPGM^f^	4.5	1.5	4 × 10^26^

a Expressed either as (*k*_ex_/*K*_d_)/*k*_non_, (*k*_cat_/*K*_m_)/*k*_non_, or (*k*_obs_/*K*_d_)/*k*_non_, where *k*_non_ is the rate constant for the corresponding spontaneous noncatalyzed reaction.

b For the proton–deuterium exchange reaction involving 5-fluorouridine 5′-monophosphate,^19^ catalytic proficiency = (*k*_ex_/*K*_d_)/*k*_non_.^54^

c For the hydride transfer reaction between NADH and dihydroxyacetone phosphate,^20^ catalytic proficiency = (*k*_cat_/*K*_m_)/*k*_non_.^55^

d For the proton transfer isomerization reaction between dihydroxyacetone phosphate and (*R*)-glyceraldehyde 3-phosphate,^18^ catalytic proficiency = (*k*_cat_/*K*_m_)/*k*_non_.^18,56^

e For the decarboxylation of orotidine 5′-monophosphate,^19^ catalytic proficiency = (*k*_cat_/*K*_m_)/*k*_non_.^19,57,58^

f For the conversion of βG1P to G6P via a βG16BP reaction intermediate (using βPGM_WT_ and βPGM_R49A_ kinetic parameters, Table 1), catalytic proficiency = (*k*_obs_/*K*_d_)/*k*_non_, where *k*_obs_ = 70 s^–1^, apparent *K*_d_ (G6P) = 9 μM, and *k*_non_ = 2.0 × 10^–20^ s^–1^ for the spontaneous noncatalyzed rate constant for phosphomonoester dianion hydrolysis.^27^

Finally, examination of the multitude of crystal structures now reported for βPGM enables a detailed illustration of the cascade of events that leads to domain closure upon hexose 1-phosphate binding. In an experiment-based animation illustrating part of the catalytic cycle (Movie S2), the open domain arrangement closes by 75% upon binding of βG1P by βPGM^P^ (Figure S2), as the hydrogen bonding relationship between the pairwise carboxamide groups of N77 and N118 lose all but one of their mediating water molecules. Direct hydrogen bond formation involving the polar side chains of S116 and N118, the side chain of R49, and replacement of the alkylammonium side chain of K117 with the backbone amide of K117 act in a concerted manner to coordinate the phosphodianion group of βG1P. Meanwhile, the hydroxyl groups attached to C2, C3, and C4 of βG1P are coordinated by several residues of the cap domain (W24, G46, S52, and K76) in an equivalent arrangement to that present in the TSA complex. Engagement of D10 into the active site to form a near-attack complex follows a rearrangement of hinge residues (D15 and T16), which brings about nucleophilic alignment and additional domain closure. The fully closed transition state conformation, compatible with proton transfer between the general acid–base and βG1P, together with phosphoryl transfer between donor and acceptor oxygen atoms, is accompanied by repositioning of a water molecule coordinated by the side chains of D10 and H20 (indicated by a gray hydrogen bond, Movie S2B). For the animation illustrating inhibition by hexose 1-phosphates stabilizing the closed inhibited βPGM complex (Movie S3), an almost identical trajectory of enzyme closure is found despite sharing only one common structure. In the inhibition trajectory, the phosphodianion group of αGal1P is coordinated in an analogous arrangement to βG1P, and rearrangement of the hinge residues, together with recruitment of D10, allows the βPGM:αGal1P complex to achieve a fully closed, near-transition state conformation. Together, the animations reveal a model of how the intrinsic binding energy of the phosphodianion group derived from the distal site stabilizes E_C_:S, irrespective of the presence of a phosphodianion group in the proximal site.

## Conclusion

The results presented establish a structural model of how enzymes that act upon phosphorylated substrates use the energy derived from the cation–phosphodianion interaction to achieve efficient catalysis on a biological time scale. Moreover, for such enzymes with high catalytic proficiencies, the intrinsic binding energy derived from the phosphodianion group in a distal site is fully utilized in stabilizing the closed active form before the adoption of the transition state. However, this catalytic proficiency mechanism risks introducing substrate inhibition to catalysis.

## Materials and Methods

### Reagents

Unless stated otherwise, reagents were purchased from Sigma-Aldrich, GE Healthcare, Melford Laboratories, or CortecNet.

### Biosynthesis of βG1P

βG1P was prepared enzymatically from maltose using maltose phosphorylase (EC 2.4.1.8). A solution of 1 M maltose was incubated overnight with 1.5 U/mL of maltose phosphorylase in a 0.5 M sodium phosphate buffer (pH 7.0) at 30 °C. βG1P production was confirmed using ^31^P NMR spectroscopy. Maltose phosphorylase (90 kDa) was removed from the solution by centrifugation using a Vivaspin (5 kDa molecular weight cut off, Sartorius), and the flow-through was used without further purification. Estimated concentrations of the components were 150 mM βG1P, 150 mM glucose, 850 mM maltose, and 350 mM P_i_.

### ^15^N-βPGM Expression and Purification

The βPGM_R49K_ and βPGM_R49A_ gene sequences were created by modifying the *pgmB* gene (encoding the βPGM_WT_ enzyme) from *Lactococcus lactis* (subspecies *lactis* IL1403; NCBI: 1114041). The βPGM_R49K_ and βPGM_R49A_ genes were generated and inserted into a pET22b(+) vector by GenScript. The βPGM_WT_, βPGM_R49K_, βPGM_R49A_, and βPGM_D170N_^50^ plasmids were transformed into *Escherichia coli* BL21(DE3) cells and expressed in defined ^15^N isotopically enriched M9 minimal media to obtain uniformly ^15^N-labeled protein.^59^ Cells were grown at 37 °C with shaking until OD_600 nm_ = 0.6, cooled at 25 °C, and induced with 0.5 mM isopropyl β-d-1-thiogalactopyranoside (IPTG) for 18 h. Cells were harvested by centrifugation at 15 000*g* for 10 min (Beckman Coulter Avanti centrifuge, Rotor: JA-14). The cell pellet was resuspended in ice-cold standard purification buffer (50 mM K^+^ HEPES (pH 7.2), 5 mM MgCl_2_, 2 mM NaN_3_, 1 mM EDTA) supplemented with cOmplete protease inhibitor cocktail and lysed by 6 × 20 s cycles of sonication (Fisherbrand Model 505 Sonic Dismembrator, 30% amplitude). The cell lysate was cleared by centrifugation at 48 000*g* for 35 min at 4 °C (Beckman Coulter Avanti centrifuge, Rotor: JA-20). The soluble fraction was filtered using a 0.22 μm syringe filter and loaded onto a DEAE-Sepharose fast flow anion-exchange column connected to an ÄKTA Prime purification system, which had been washed previously with 1 M NaOH and 6 M guanidinium chloride and equilibrated with five column volumes of standard purification buffer. Bound proteins were eluted using a gradient of 0 to 50% standard purification buffer containing 1 M NaCl over 300 mL. Fractions containing βPGM were identified by SDS-PAGE and concentrated to a 5–10 mL volume using centrifugation in a Vivaspin (10 kDa molecular weight cut off, Sartorius) at 3400*g* and 4 °C (Thermo Scientific Heraeus Labofuge 400 R). The concentrated protein sample was loaded onto a prepacked Hiload 26/600 Superdex 75 size-exclusion column connected to an ÄKTA Prime purification system, which had been washed previously with degassed 1 M NaOH and equilibrated with three column volumes of degassed standard purification buffer supplemented with 1 M NaCl. Proteins were eluted using this buffer, and fractions containing βPGM were checked for purity, pooled, and buffer-exchanged and concentrated (to 1 mM) into standard purification buffer using a Vivaspin (10 kDa molecular weight cut off, Sartorius). The protein concentration was measured using a NanoDrop One^C^ spectrophotometer (Thermo Scientific; βPGM molecular weight = 24.2 kDa, extinction coefficient = 19 940 M^–1^ cm^–1^) and stored at −20 °C. All kinetic assays, NMR spectroscopy, and X-ray crystallography experiments were performed using uniformly ^15^N-labeled βPGM.

### NMR Analysis of Substrate-Free βPGM

^1^H^15^N-TROSY NMR spectra of substrate-free βPGM_WT_, substrate-free βPGM_R49K_, and substrate-free βPGM_R49A_ were acquired at 298 K using a Bruker 500 MHz Avance III HD spectrometer equipped with a 5 mm QCI-F cryoprobe and *z*-axis gradients. Samples contained 1 mM βPGM in standard NMR buffer (50 mM K^+^ HEPES (pH 7.2), 5 mM MgCl_2_, 2 mM NaN_3_, with 10% (v/v) ^2^H_2_O and 2 mM trimethylsilyl propionate (TSP)). Typically, ^1^H^15^N-TROSY NMR spectra were accumulations of 32 transients with 256 increments and spectral widths of 32–36 ppm centered at 120 ppm in the indirect ^15^N-dimension. Experiments were processed using TopSpin (Bruker), and NMR figures were prepared using FELIX (Felix NMR, Inc.). ^1^H chemical shifts were referenced relative to the internal TSP signal resonating at 0.0 ppm, and ^15^N chemical shifts were referenced indirectly using nuclei-specific gyromagnetic ratios.

### NMR Analysis of βPGM TSA Complexes

^1^H^15^N-TROSY NMR spectra of βPGM_WT_:AlF_4_:G6P, βPGM_R49K_:AlF_4_:G6P, βPGM_R49A_:AlF_4_:G6P, βPGM_WT_:MgF_3_:G6P, βPGM_R49K_:MgF_3_:G6P, and βPGM_R49A_:MgF_3_:G6P TSA complexes were acquired at 298 K as described above using a Bruker 500 MHz Avance III HD spectrometer equipped with a 5 mm QCI-F cryoprobe and *z*-axis gradients. Samples contained 0.5–1.5 mM βPGM in standard NMR buffer (50 mM K^+^ HEPES (pH 7.2), 5 mM MgCl_2_, 2 mM NaN_3_, with 10% (v/v) ^2^H_2_O and 2 mM TSP), together with 15 mM NaF, (3 mM AlCl_3_), and 20 mM G6P. One-dimensional ^19^F NMR spectra were acquired without proton decoupling and were processed with 10 Hz Lorentzian apodization using TopSpin (Bruker). ^19^F chemical shifts were referenced indirectly using nuclei-specific gyromagnetic ratios.

### Measurement of Apparent Dissociation Constants by ^1^H NMR Spectroscopy

The apparent dissociation constants for G6P (apparent *K*_d_ (G6P)) for the βPGM_R49K_:AlF_4_:G6P and βPGM_R49A_:AlF_4_:G6P TSA complexes were determined at 298 K using a Bruker Neo 800 MHz spectrometer equipped with a 5 mm TCI cryoprobe and *z*-axis gradients. A solution of 360–400 mM G6P in standard NMR buffer was titrated serially into separate solutions containing either 0.5 mM βPGM_R49K_ or 0.5 mM βPGM_R49A_ prepared in standard NMR buffer supplemented with 15 mM NaF and 3 mM AlCl_3_. The titrations were monitored by the acquisition of one-dimensional ^1^H NMR spectra and were processed using TopSpin (Bruker). The changing intensity of the well-resolved indole resonance of residue W24 (acting as a reporter for G6P binding and adoption of the closed TSA complex in slow exchange) was fitted using a nonlinear least-squares fitting algorithm corrected for dilution effects to determine apparent *K*_d_ (G6P) values.

### Reaction Kinetics Monitored Using ^31^P NMR Spectroscopy

Reaction kinetics of βPGM_WT_, βPGM_R49K_, and βPGM_R49A_ were followed at 298 K using a Bruker 500 MHz Avance III HD spectrometer (operating at 202.48 MHz for ^31^P) equipped with a 5 mm Prodigy BBO cryoprobe. One-dimensional ^31^P NMR spectra recorded without proton decoupling were acquired within 1 min with 16 transients and a 2 s recycle delay to give signal-to-noise ratios for 10 mM βG1P of greater than 100:1. The equilibration of 10 mM βG1P with G6P by either 0.05 μM βPGM_WT_, 0.5 μM βPGM_R49K_, or 1.0 μM βPGM_R49A_ was measured in standard kinetic buffer (200 mM K^+^ HEPES (pH 7.2), 5 mM MgCl_2_, 2 mM NaN_3_) with the addition of 10% (v/v) ^2^H_2_O and 2 mM TSP. The reaction was initiated by 20 mM AcP and timed immediately after its addition. The reaction was monitored by the acquisition of consecutive ^31^P NMR experiments. Spectra were processed using TopSpin (Bruker), and normalized integral values of the G6P peak following baseline correction and 2 Hz Lorentzian apodization were plotted against time to give kinetic profiles. The linear steady-state portion of the data was fitted using a linear least-squares fitting algorithm to derive a reaction rate, which was multiplied by the initial βG1P concentration and normalized by the enzyme concentration to obtain the observed catalytic rate constant (*k*_obs_).

### Reaction Kinetics Monitored by Glucose 6-Phosphate Dehydrogenase Coupled Assay

Kinetic assays for βPGM_WT_, βPGM_R49K_, and βPGM_R49A_ were conducted at 294 K using a FLUOstar OMEGA microplate reader and the BMG LABTECH Reader Control Software (version 5.11; BMG Labtech) in standard kinetic buffer (200 mM K^+^ HEPES (pH 7.2), 5 mM MgCl_2_, and 1 mM NaN_3_) in a 200 μL reaction volume. The rate of G6P production was measured indirectly using a glucose 6-phosphate dehydrogenase (G6PDH) coupled assay, in which G6P is oxidized and concomitant NAD^+^ reduction is monitored by the increase in absorbance at 340 nm (NADH extinction coefficient = 6220 M^–1^ cm^–1^). βPGM_WT_, βPGM_R49K_, and βPGM_R49A_ concentrations were determined using a NanoDrop One^C^ spectrophotometer (Thermo Scientific) and diluted accordingly. Reactions were conducted in triplicate and were initiated by the addition of 20 mM AcP (10 mM AcP for βPGM_WT_) to solutions containing 1 mM NAD^+^ (0.5 mM NAD^+^ for βPGM_WT_) and 5 units mL^–1^ of G6PDH, together with variable concentrations of βG1P (5, 15, 35, 50, 70, 100, 160, 230, 330 μM) and either 5 nM βPGM_WT_, 60 nM βPGM_R49K_, or 60 nM βPGM_R49A_. The linear steady-state portion of G6P production was fitted using a linear least-squares fitting algorithm to determine the reaction velocity (*v*) at each βG1P concentration. Data were subsequently fitted to the standard Michaelis–Menten equation using an in-house python nonlinear least-squares fitting algorithm to derive apparent *k*_cat_ and apparent *K*_m_ (βG1P) values. Data were also fitted to a linear equation to derive *k*_cat_/*K*_m_ ratios. Errors were estimated using a python bootstrap resampling protocol and are presented at one standard deviation.

### X-ray Crystallography

Frozen aliquots of substrate-free βPGM in standard native buffer (50 mM K^+^ HEPES (pH 7.2), 5 mM MgCl_2_ and 1 mM NaN_3_) were thawed on ice and centrifuged briefly to pellet insoluble material. Crystals of the βPGM:AlF_4_:G6P TSA complexes were obtained from a solution of substrate-free βPGM containing 20 mM NaF, 5 mM AlCl_3_, and 10 mM G6P. Crystals of the βPGM:MgF_3_:G6P TSA complexes were obtained from a solution of substrate-free βPGM containing 20 mM NaF and 10 mM G6P. Crystals of the βPGM_D170N_:βG1P complex were obtained from a solution of substrate-free βPGM_D170N_ containing 20 mM NaF and 10 mM G6P. Crystals of the βPGM_WT_:P_i_ complex were obtained from a solution of substrate-free βPGM_WT_ containing 10 mM glucose, 10 mM sodium phosphate, and 15 mM NaF. Solutions were adjusted to a final protein concentration of 0.4–0.6 mM, incubated for ∼10 min and mixed 1:1 with precipitant (26–30% (w/v) PEG 4000, 200 mM sodium acetate, and 100 mM tris-HCl (pH 7.5)). Crystals were grown at 290 K by hanging-drop vapor diffusion using a 2 μL drop suspended on a siliconized glass coverslip above a 700 μL well. Thin plate, small needle, or rod-shaped crystals grew typically over several days. Crystals were harvested using a mounted LithoLoop (Molecular Dimensions Ltd.) and were cryo-protected in their mother liquor containing an additional 25% (v/v) ethylene glycol prior to plunging into liquid nitrogen. Diffraction data were collected at 100 K on the MX beamlines at the Diamond Light Source (DLS), Oxfordshire, United Kingdom and on beamline ID14-2 at the European Synchrotron Radiation Facility (ESRF), Grenoble, France. At the DLS, data were processed using the xia2 pipeline,^60^ whereas at the ESRF, data were processed with iMOSFLM.^61^ Resolution cutoffs were applied using either CC-half values or by consideration of the <*I*/σ(*I*)> and *R*_merge_ values. Structures were determined by molecular replacement with MolRep^62^ using previously deposited βPGM structures with the most appropriate cap and core domain relationship as search models. Model building was carried out in COOT,^63^ and a restrained refinement with either isotropic temperature factors (resolution >1.5 Å) or anisotropic temperature factors (resolutions <1.5 Å) was performed using REFMAC5^64^ in the CCP4i suite.^65^ Ligands were not included until the final stages of refinement to avoid biasing Fourier maps. Structure validation was carried out in COOT and MolProbity;^66^ superpositions were generated using PyMOL (The PyMOL Molecular Graphics System, version 1.8/2.2 Schrödinger, LLC). Maps were generated using FFT,^67^ and domain movements were calculated using DynDom.^68^

### Animations

For the pairwise active site animations, png files of the corresponding βPGM_WT_:AlF_4_:G6P, βPGM_R49K_:AlF_4_:G6P, βPGM_R49A_:AlF_4_:G6P, βPGM_WT_:MgF_3_:G6P, βPGM_R49K_:MgF_3_:G6P, and βPGM_R49A_:MgF_3_:G6P TSA complexes were generated using PyMOL. Pairs of images were combined to form animated gif files. The following crystal structures were used to generate the animation illustrating part of the βPGM catalytic cycle: the βPGM_WT_:BeF_3_ complex (PDB 2WFA)^36^ as a mimic of open βPGM_WT_^P^, together with βG1P docked in the active site, the βPGM_WT_:P_i_ complex (PDB 6H93, chain B) with P_i_ replaced by βG1P as a mimic of a slightly closed βPGM_WT_^P^:βG1P complex, the βPGM_D10N_:AlF_4_:H_2_O:βG1P complex (PDB 5O6R)^38^ as a mimic of the βPGM_WT_^P^:βG1P near attack complex, the βPGM_WT_:MgF_3_:βG1 phosphonate TSA complex (PDB 4C4R)^37^ as a mimic of a fully closed, near-transition state complex, the βPGM_D10N_:βG16BP complex (PDB 5OK0)^38^ as a mimic of the βPGM_WT_:βG16BP near attack complex, the βPGM_WT_:P_i_ complex (PDB 6H93, chain B) with P_i_ replaced by βG16BP, and the βPGM_WT_:BeF_3_ complex (PDB 2WFA)^36^ as a mimic of open βPGM_WT_ (with the BeF_3_ moiety removed) along with βG16BP docked in the active site. The following crystal structures were used to generate the animation illustrating inhibition by hexose 1-phosphates facilitating the closure of nonphosphorylated βPGM: substrate-free βPGM_WT_ (PDB 2WHE)^35^ with αGal1P docked in the open active site, the βPGM_WT_:P_i_ complex (PDB 6H93, chain B) with P_i_ replaced by αGal1P, the βPGM_WT_:αGal1P complex (PDB 1Z4O, chain B)^52^ as a model of a near attack complex, and the βPGM_WT_:αGal1P complex (PDB 1Z4O, chain A)^52^ as a model of a fully closed, near-transition state complex. The PDB files were edited accordingly to provide a systematic atom nomenclature across all the complexes involved. The docking of either βG1P, βG16BP, or αGal1P within the active site of βPGM_WT_ was performed using PyMOL. Morphing between pairs of PDB files in the trajectory was achieved with Cartesian interpolation using LSQMAN (G. J. Kleywegt, Uppsala Software Factory). Rendering of the subsequent PDB file trajectory and generation of the corresponding png files was achieved using PyMOL. Images comprising both the animation illustrating part of the βPGM catalytic cycle and the animation illustrating inhibition by hexose 1-phosphates facilitating the closure of nonphosphorylated βPGM were combined to form separate animated gif files. All animated gif files were then converted to mp4 files using Adobe Photoshop.

## Supporting Information Available

Figures containing ^1^H^15^N TROSY NMR spectra; chemical shift perturbation analysis; pairwise interdomain rotation angles between βPGM structures; pairwise RMSD values between superpositions of βPGM structures; determination of apparent *K*_d_ (G6P) values; βPGM reaction kinetics; active sites of βPGM complexes showing difference density; tables containing X-ray data collection, data processing, and refinement statistics (PDF)
Animation comparing the βPGM_R49K_:AlF_4_:G6P and βPGM_WT_:AlF_4_:G6P TSA complexes (MP4)
Animation comparing the βPGM_R49A_:AlF_4_:G6P and βPGM_WT_:AlF_4_:G6P TSA complexes (MP4)
Animation comparing the βPGM_R49K_:MgF_3_:G6P and βPGM_WT_:MgF_3_:G6P TSA complexes (MP4)
Animation comparing the βPGM_R49A_:MgF_3_:G6P and βPGM_WT_:MgF_3_:G6P TSA complexes (MP4)
Animation illustrating part of the βPGM catalytic cycle; view showing the full enzyme (MP4)
Animation illustrating part of the βPGM catalytic cycle; view focusing on the distal site (MP4)
Animation illustrating the inhibition by hexose 1-phosphates facilitating the closure of nonphosphorylated βPGM; view showing the full enzyme (MP4)
Animation illustrating the inhibition by hexose 1-phosphates facilitating the closure of nonphosphorylated βPGM; view focusing (MP4)

## Accession Codes

Data supporting the findings of this manuscript are available from the corresponding author upon request. The atomic coordinates and structure factors have been deposited in the Protein Data Bank (www.rcsb.org) with the following codes: substrate-free βPGM_D170N_ (PDB 6HDF), βPGM_D170N_:βG1P complex (PDB 6HDG), βPGM_WT_:P_i_ complex (PDB 6H93), substrate-free βPGM_R49K_ (PDB 6HDH), substrate-free βPGM_R49A_ (PDB 6HDI), βPGM_WT_:AlF_4_:G6P TSA complex (PDB 2WF6), βPGM_R49K_:AlF_4_:G6P TSA complex (PDB 6HDJ), βPGM_R49A_:AlF_4_:G6P TSA complex (PDB 6HDK), βPGM_R49K_:MgF_3_:G6P TSA complex (PDB 6HDL), and βPGM_R49A_:MgF_3_:G6P TSA complex (PDB 6HDM).
