# Supplementary material for: An Enzyme with High Catalytic Proficiency Utilizes Distal Site Substrate Binding Energy to Stabilize the Closed State but at the Expense of Substrate Inhibition
Source: ACS Catal. 2022 Feb 22;12(5):3149–64. doi: 10.1021/acscatal.1c05524 (PMC9171722; doi:10.1021/acscatal.1c05524)
Supplement: Supplementary file 1 — cs1c05524_si_001.pdf [file cs1c05524_si_001.pdf]

SUPPORTING INFORMATION

An Enzyme with High Catalytic Proficiency Utilizes Distal Site Substrate Binding Energy to Stabilize the Closed State but at the Expense of Substrate Inhibition

Angus J. Robertson,[a,#] *F. Aaron Cruz-Navarrete,*[a,#] *Henry P. Wood,*[a] *Nikita Vekaria,*[b] *Andrea M. Hounslow,*[a] *Claudine Bisson,*[a] *Matthew J. Cliff,*[b] *Nicola J. Baxter,*[a,b] *and Jonathan P. Waltho*[a,b,*]

[a] School of Biosciences, The University of Sheffield, Sheffield, S10 2TN, United Kingdom

[b] Manchester Institute of Biotechnology and Department of Chemistry, The University of Manchester, Manchester, M1 7DN, United Kingdom

[*] Corresponding author: Prof. Jonathan P. Waltho. Email: j.waltho@sheffield.ac.uk

[#] These authors contributed equally

This PDF file includes:

Figures S1 to S9
Table S1
Legends for Movies S1 to S3

Other supplementary materials for this manuscript include the following:

Movies S1 to S3

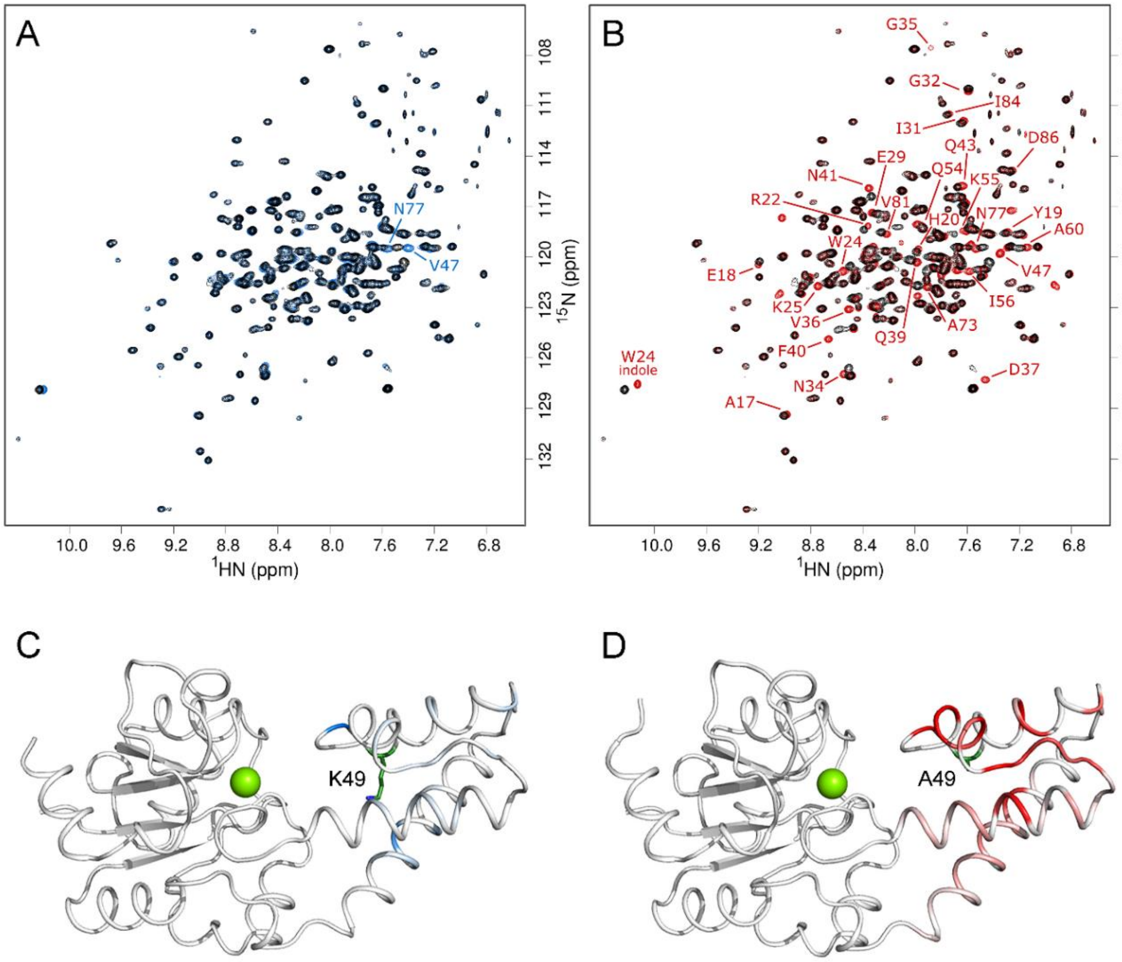

Figure S1. Solution behavior of substrate-free βPGM. (A–B) Pairwise overlays of $^{1}H^{15}N$-TROSY NMR spectra for (A) substrate-free βPGM_{WT} (black) and substrate-free βPGM_{R49K} (blue), and (B) substrate-free βPGM_{WT} (black) and substrate-free βPGM_{R49A} (red), acquired in standard NMR buffer. Backbone amide peaks for βPGM_{R49K} and βPGM_{R49A} that shift their positions relative to βPGM_{WT} are labeled. There is high degree of correspondence between βPGM_{WT} and βPGM_{R49K}, indicating that the R49K substitution does not have a significant impact on the protein fold (residues V47 and N77 are within 5 Å of K49). In marked contrast, small but widespread differences in peak positions between βPGM_{WT} and βPGM_{R49A} show that the R49A substitution has a moderate effect on the solution properties of the helical cap domain (T16–V87). *cis-trans* isomerization of the K145-P146 peptide bond which is observed in βPGM_{WT} [28] is also present for βPGM_{R49K} and βPGM_{R49A}, and results in the population of two conformers in slow exchange (~70% *cis*-P146 and ~30% *trans*-P146). Additionally, *ca.* six peaks are present in βPGM_{R49A} that are absent in βPGM_{WT} due to backbone conformational exchange on the millisecond timescale.[28] This observation indicates that residue A49 in βPGM_{R49A} abolishes the intermediate exchange dynamic that residue R49 propagates in βPGM_{WT}. (C–D) Weighted chemical shift changes for substrate-free βPGM_{R49K} and substrate-free βPGM_{R49A} with respect to substrate-free βPGM_{WT} are calculated for the backbone amide group of each residue as $\Delta\delta = [(\delta_{HN\text{-}X} - \delta_{HN\text{-}Y})^2 + (0.13 \times (\delta_{N\text{-}X} - \delta_{N\text{-}Y}))^2]^{1/2}$, where X and Y are the two species being compared. (C) Crystal structure of βPGM_{R49K} (PDB 6HDH, chain A) showing residues of the cap domain with 0.00 ppm < $\Delta\delta \leq 0.11$ ppm colored in shades of blue for the βPGM_{WT} and βPGM_{R49K} pairwise comparison. Mg_{cat}^{2+} (green sphere) and residue K49 (green sticks) are highlighted. (D) Crystal structure of βPGM_{R49A} (PDB 6HDI, chain A) showing residues of the cap domain with 0.00 ppm < $\Delta\delta \leq 0.16$ ppm colored in shades of red for the βPGM_{WT} and βPGM_{R49A} pairwise comparison. Mg_{cat}^{2+} (green sphere) and residue A49 (green sticks) are highlighted.

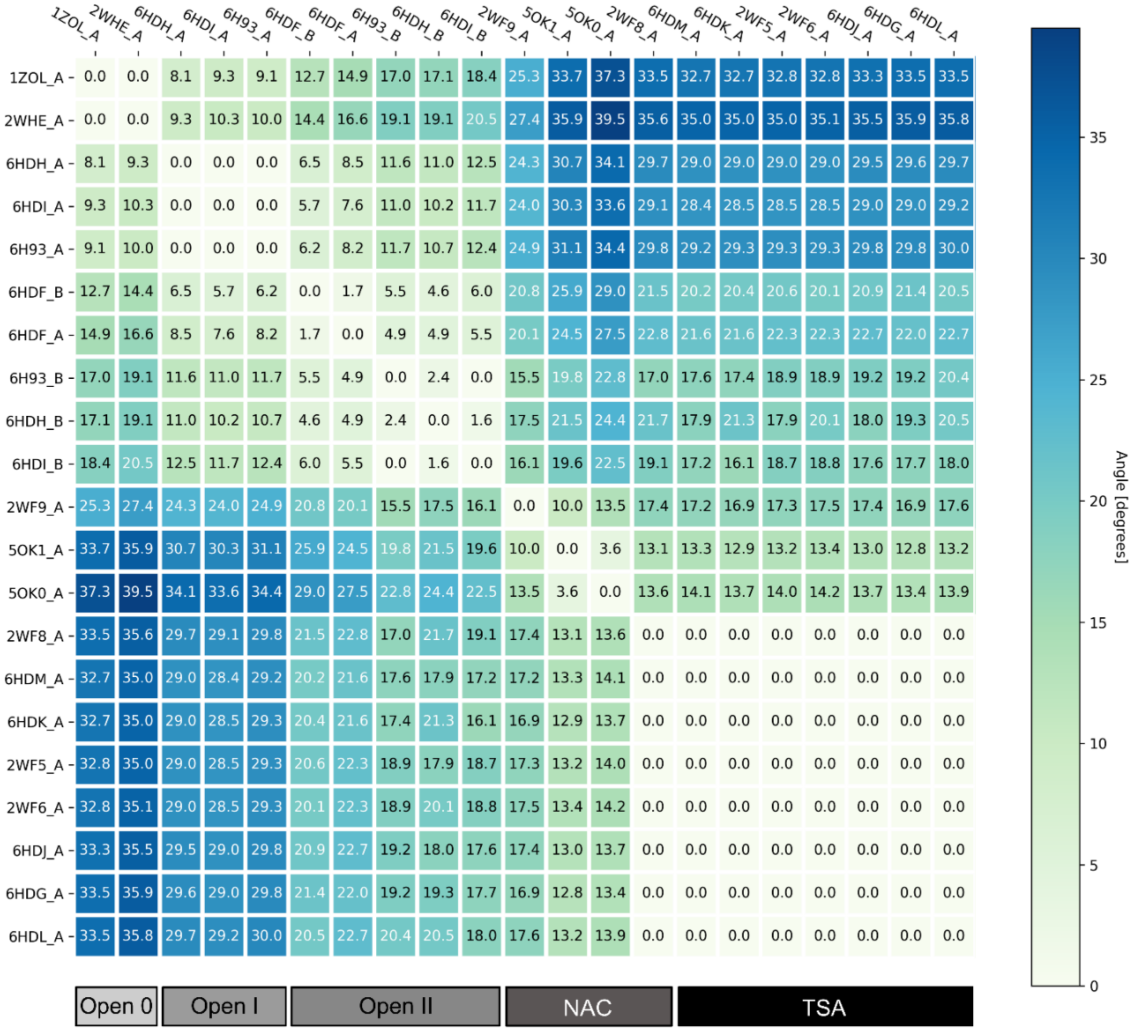

Figure S2. The closure angle (°) describing cap domain movement through rotation at the interdomain hinge between pairs of βPGM crystal structures determined using DynDom.[68] Comparisons where no dynamic domains were found by the algorithm are denoted with a rotation angle of 0.0° with an upper bound of 0.2°. Crystal structures of substrate-free βPGM and βPGM complexes, together with their corresponding PDB identification codes are listed as follows: substrate-free βPGM_{WT} (PDB 1ZOL)[31] substrate-free βPGM_{WT} (PDB 2WHE),[35] substrate-free βPGM_{R49K} (PDB 6HDH), substrate-free βPGM_{R49A} (PDB 6HDI), βPGM_{WT}:P_i complex (PDB 6H93), substrate-free βPGM_{D170N} (PDB 6HDF), βPGM_{WT}:BeF_3:G6P complex (PDB 2WF9),[36] βPGM_{D10N}:βG16BP complex (PDB 5OK1),[38] βPGM_{D10N}:βG16BP complex (PDB 5OK0),[38] βPGM_{WT}:BeF_3:βG1P complex (PDB 2WF8),[36] βPGM_{R49A}:MgF_3:G6P complex (PDB 6HDM), βPGM_{R49A}:AlF_4:G6P complex (PDB 6HDK), βPGM_{WT}:MgF_3:G6P complex (PDB 2WF5),[35] βPGM_{WT}:AlF_4:G6P complex (PDB 2WF6), βPGM_{R49K}:AlF_4:G6P complex (PDB 6HDJ), βPGM_{D170N}:βG1P complex (PDB 6HDG), and βPGM_{R49K}:MgF_3:G6P complex (PDB 6HDL). PDB identification codes containing suffixes _A and _B denote chain A and chain B, respectively for monomers of the asymmetric unit. Crystal structures have been categorized as open with three clusters of interdomain closure angle, near attack complexes (NAC) or transition state analogue (TSA) complexes and are indicated by bars.

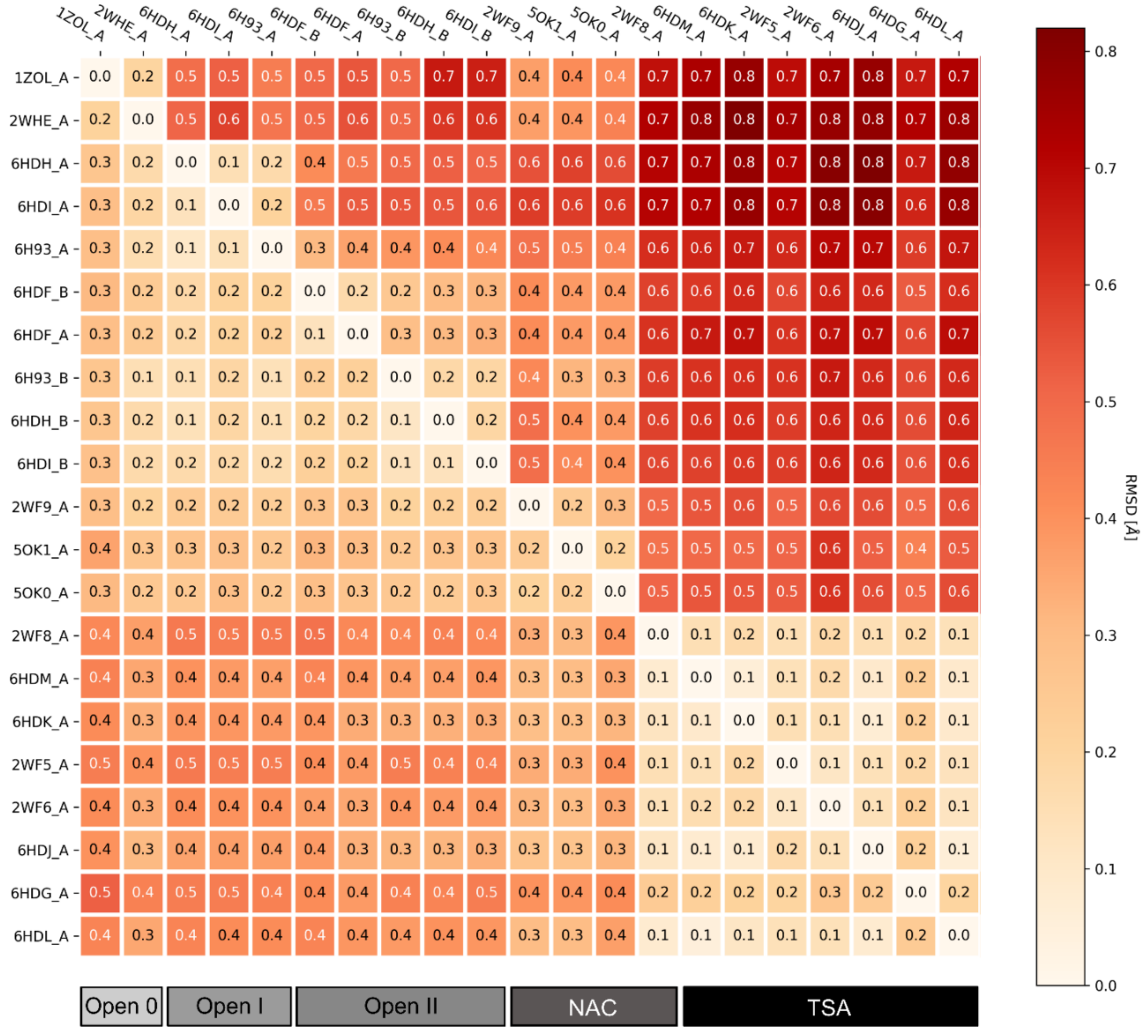

Figure S3. Non-H atom RMSD values (Å) for cap domain and core domain alignments between pairs of βPGM crystal structures determined using PyMOL (The PyMOL Molecular Graphics System, version 1.8/2.2 Schrödinger, LLC). The upper right pseudo-triangular matrix indicates pairwise RMSD values for cap domain (T16–V87) alignments, while the lower left pseudo-triangular matrix indicates pairwise RMSD values for core domain (M1–D15, S88–K221) alignments. A greater level of perturbation is observed for residues of the cap domain in pairwise comparisons involving structures with different closure angles. Crystal structures of substrate-free βPGM and βPGM complexes, together with their corresponding PDB identification codes are listed as follows: substrate-free βPGM$_{WT}$ (PDB 1ZOL),[31] substrate-free βPGM$_{WT}$ (PDB 2WHE),[35] substrate-free βPGM$_{R49K}$ (PDB 6HDH), substrate-free βPGM$_{R49A}$ (PDB 6HDI), βPGM$_{WT}$:P$_i$ complex (PDB 6H93), substrate-free βPGM$_{D170N}$ (PDB 6HDF), βPGM$_{WT}$:BeF$_3$:G6P complex (PDB 2WF9),[36] βPGM$_{D10N}$:βG16BP complex (PDB 5OK1),[38] βPGM$_{D10N}$:βG16BP complex (PDB 5OK0),[38] βPGM$_{WT}$:BeF$_3$:βG1P complex (PDB 2WF8),[36] βPGM$_{R49A}$:MgF$_3$:G6P complex (PDB 6HDM), βPGM$_{R49A}$:AlF$_4$:G6P complex (PDB 6HDK), βPGM$_{WT}$:MgF$_3$:G6P complex (PDB 2WF5),[35] βPGM$_{WT}$:AlF$_4$:G6P complex (PDB 2WF6), βPGM$_{R49K}$:AlF$_4$:G6P complex (PDB 6HDJ), βPGM$_{D170N}$:βG1P complex (PDB 6HDG), and βPGM$_{R49K}$:MgF$_3$:G6P complex (PDB 6HDL). PDB identification codes containing suffixes _A and _B denote chain A and chain B, respectively for monomers of the asymmetric unit. Crystal structures have been categorized as open with three clusters of interdomain closure angle, near attack complexes (NAC) or transition state analogue (TSA) complexes and are indicated by bars.

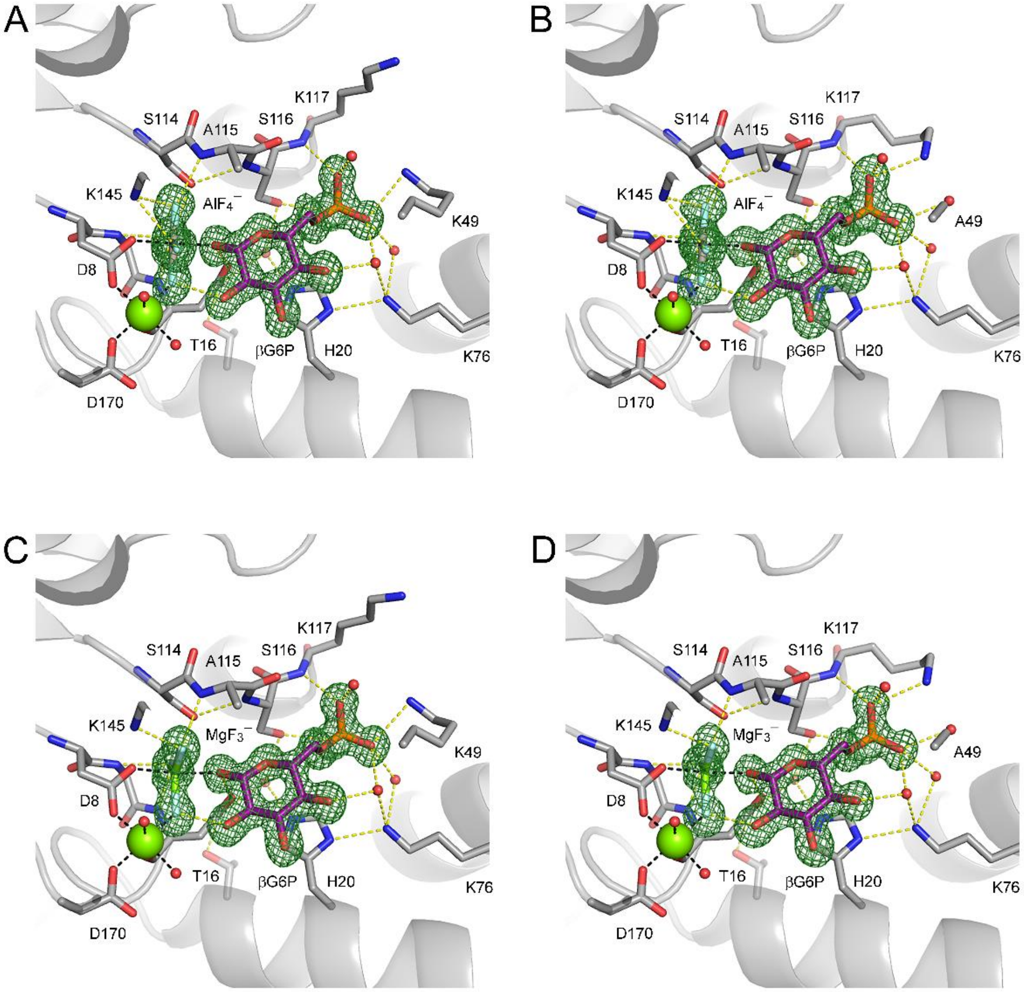

Figure S4. Difference density and active site details of the βPGM:AlF_4:G6P and βPGM:MgF_3:G6P TSA complexes. The active sites of (A) βPGM_{R49K}:AlF_4:G6P complex (PDB 6HDJ), (B) βPGM_{R49A}:AlF_4:G6P complex (PDB 6HDK), (C) βPGM_{R49K}:MgF_3:G6P complex (PDB 6HDL), and (D) βPGM_{R49A}:MgF_3:G6P complex (PDB 6HDM). Selected residues (sticks), together with the square-planar AlF_4^- moiety (dark gray and light blue sticks), the trigonal-planar MgF_3^- moiety (green and light blue sticks), βG6P (purple carbon atoms), structural waters (red spheres), and Mg_{cat}^{2+} (green sphere) are illustrated. Yellow dashes indicate hydrogen bonds and black dashes show metal ion coordination. Difference density (Fo – Fc, green mesh) is contoured at 3σ and was generated following ligand omission from the final structures. The side chain of residue N118, which coordinates one of the phosphodianion oxygen atoms of G6P equivalently in the TSA complexes, has been omitted for clarity.

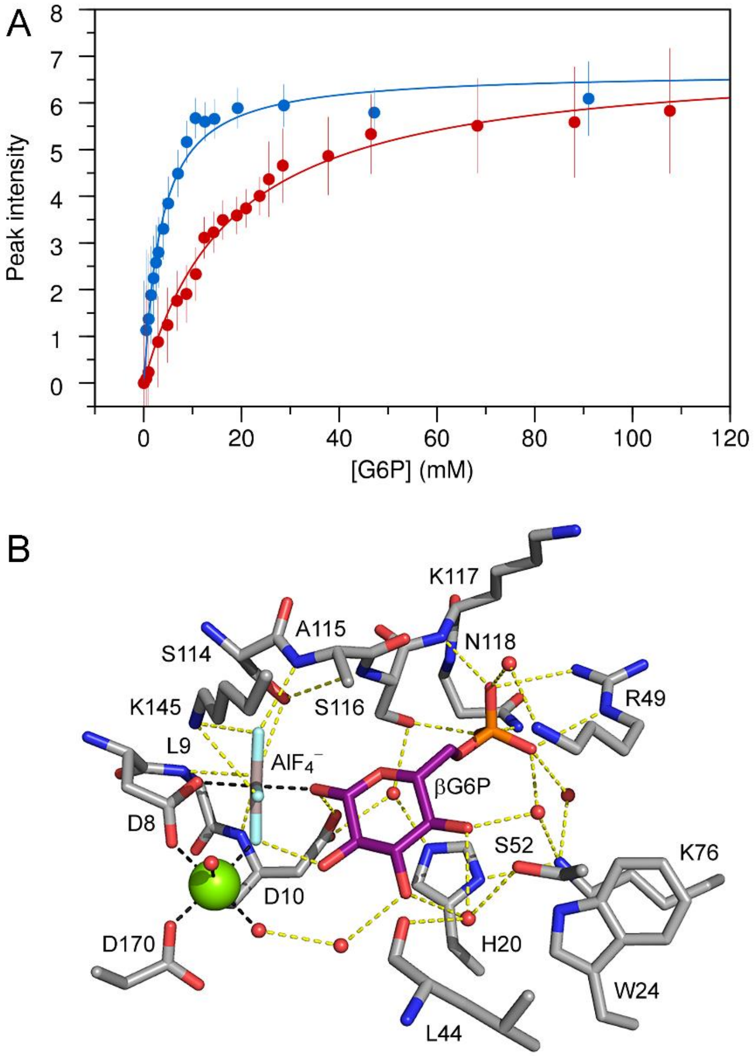

Figure S5. Determination of the apparent K_d (G6P) values for the βPGM_{R49K}:AlF_4:G6P and βPGM_{R49A}:AlF_4:G6P TSA complexes, monitored using one-dimensional ^1H NMR spectroscopy. (A) A solution of 360–400 mM G6P was titrated serially into 0.5 mM βPGM_{R49K} (blue circles) or 0.5 mM βPGM_{R49A} (red circles) prepared in standard NMR buffer supplemented with 15 mM NaF and 3 mM $AlCl_3$. The changing intensity of the well-resolved indole resonance of residue W24 (acting as a reporter for G6P binding and adoption of the closed TSA complex in slow exchange) was fitted to determine the apparent K_d (G6P) value for the βPGM_{R49K}:AlF_4:G6P TSA complex (apparent K_d (G6P) = 3.0 ± 0.4 mM) and the βPGM_{R49A}:AlF_4:G6P TSA complex (apparent K_d (G6P) = 18 ± 1 mM). Vertical error bars indicate estimated errors in the measurement of peak intensities. (B) The active site details of the βPGM_{WT}:AlF_4:G6P TSA complex (PDB 2WF6) showing the proximity of residue W24 to G6P. Selected residues (sticks), together with the square-planar AlF_4^- moiety (dark gray and light blue sticks), βG6P (purple carbon atoms), structural waters (red spheres), and Mg_{cat}^{2+} (green sphere) are illustrated. Yellow dashes indicate hydrogen bonds and black dashes show metal ion coordination. In substrate-free βPGM_{WT}, $W24_{indole}$: δ_{HN} = 10.24 ppm, δ_N = 127.63 ppm and in the βPGM_{WT}:AlF_4:G6P TSA complex, $W24_{indole}$: δ_{HN} = 10.49 ppm, δ_N = 127.75 ppm. In substrate-free βPGM_{R49K}, $W24_{indole}$: δ_{HN} = 10.20 ppm, δ_N = 127.89 ppm and in the βPGM_{R49K}:AlF_4:G6P TSA complex, $W24_{indole}$: δ_{HN} = 10.55 ppm, δ_N = 128.10 ppm. In substrate-free βPGM_{R49A}, $W24_{indole}$: δ_{HN} = 10.13 ppm, δ_N = 127.56 ppm and in the βPGM_{R49A}:AlF_4:G6P TSA complex, $W24_{indole}$: δ_{HN} = 10.46 ppm, δ_N = 127.84 ppm.

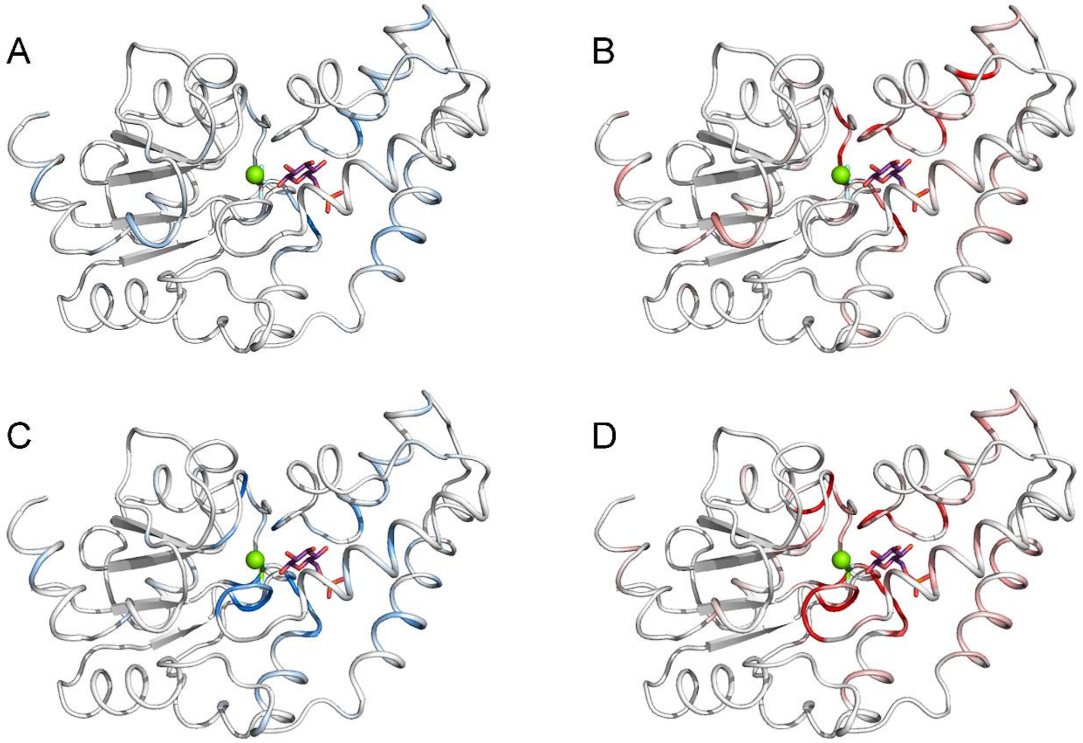

Figure S6. Chemical shift perturbations arising from R49 side chain substitution in the βPGM:AlF_4:G6P and βPGM:MgF_3:G6P TSA complexes. (A–D) Weighted chemical shift changes of the backbone amide group are calculated for each residue as: $\Delta\delta = [(\delta_{HN\text{-}X} - \delta_{HN\text{-}Y})^2 + (0.13 \times (\delta_{N\text{-}X} - \delta_{N\text{-}Y}))^2]^{1/2}$, where X and Y are the two species being compared. (A) Crystal structure of the βPGM_{R49K}:AlF_4:G6P TSA complex (PDB 6HDJ) showing residues with 0.0 ppm < Δδ ≤ 0.3 ppm between the βPGM_{R49K}:AlF_4:G6P and βPGM_{WT}:AlF_4:G6P TSA complexes colored in shades of blue. (B) Crystal structure of the βPGM_{R49A}:AlF_4:G6P TSA complex (PDB 6HDK) showing residues with 0.0 ppm < Δδ ≤ 0.5 ppm between the βPGM_{R49A}:AlF_4:G6P and βPGM_{WT}:AlF_4:G6P TSA complexes colored in shades of red. (C) Crystal structure of the βPGM_{R49K}:MgF_3:G6P TSA complex (PDB 6HDL) showing residues with 0.0 ppm < Δδ ≤ 0.4 ppm between the βPGM_{R49K}:MgF_3:G6P and βPGM_{WT}:MgF_3:G6P TSA complexes colored in shades of blue. (D) Crystal structure of the βPGM_{R49A}:MgF_3:G6P TSA complex (PDB 6HDM) showing residues with 0.0 ppm < Δδ ≤ 0.5 ppm between the βPGM_{R49A}:MgF_3:G6P and βPGM_{WT}:MgF_3:G6P TSA complexes colored in shades of red. The small magnitude (0.1–0.5 ppm) of the Δδ values indicates that the extent of perturbation across the active site due to R49 side chain substitution in a fully closed, near-transition state conformation is not substantial. The square-planar AlF_4^- moiety (dark gray and light blue sticks), the trigonal-planar MgF_3^- moiety (green and light blue sticks), βG6P (purple carbon atoms), and Mg_{cat}^{2+} (green sphere) are illustrated.

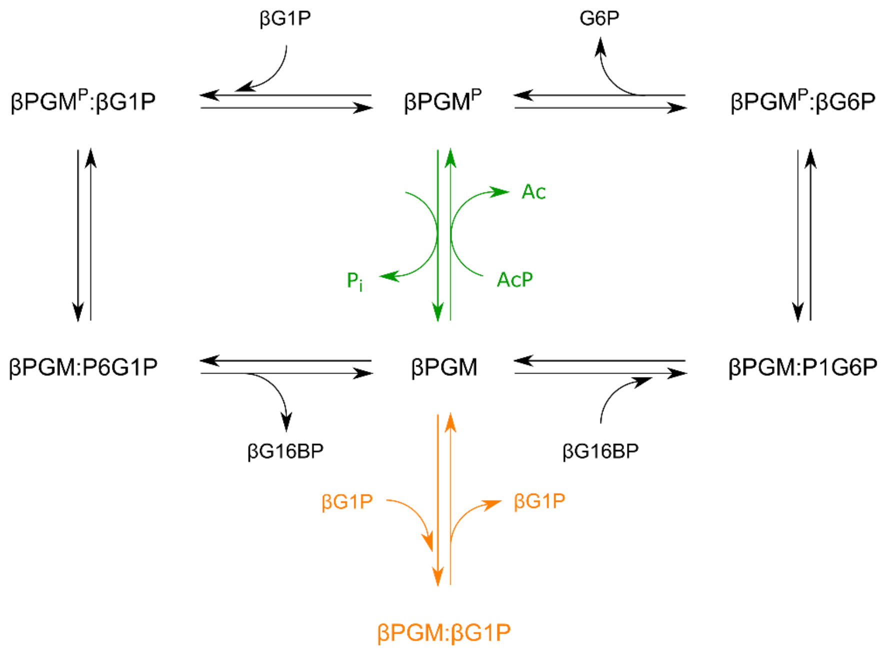

Figure S7. βPGM catalytic cycle operating in the ^{31}P NMR time-course experiments and in the coupled assay. Recombinant substrate-free βPGM exists in the nonphosphorylated form, since the half-life of βPGM^{P} is ~30 s.[34] Addition of excess acetyl phosphate (AcP, green) phosphorylates βPGM generating βPGM^{P}, but this process is not particularly efficient.[28] βPGM^{P} now catalyzes the isomerization of βG1P to G6P via a βG16BP reaction intermediate (anticlockwise reaction scheme, black). The two intermediate complexes are labeled βPGM:P6G1P and βPGM:P1G6P to explicitly denote the orientation of βG16BP bound in the active site. Moreover, binding of βG1P to substrate-free βPGM generates an inhibited βPGM:βG1P complex (orange), with a fully closed, near-transition state conformation. At early reaction times, the predominance of, and competition between, the AcP-mediated generation of βPGM^{P} and formation of the inhibited βPGM:βG1P complex results in a lag phase, which is only alleviated when the concentration of βG16BP produced by βPGM is sufficiently elevated to outcompete these processes.

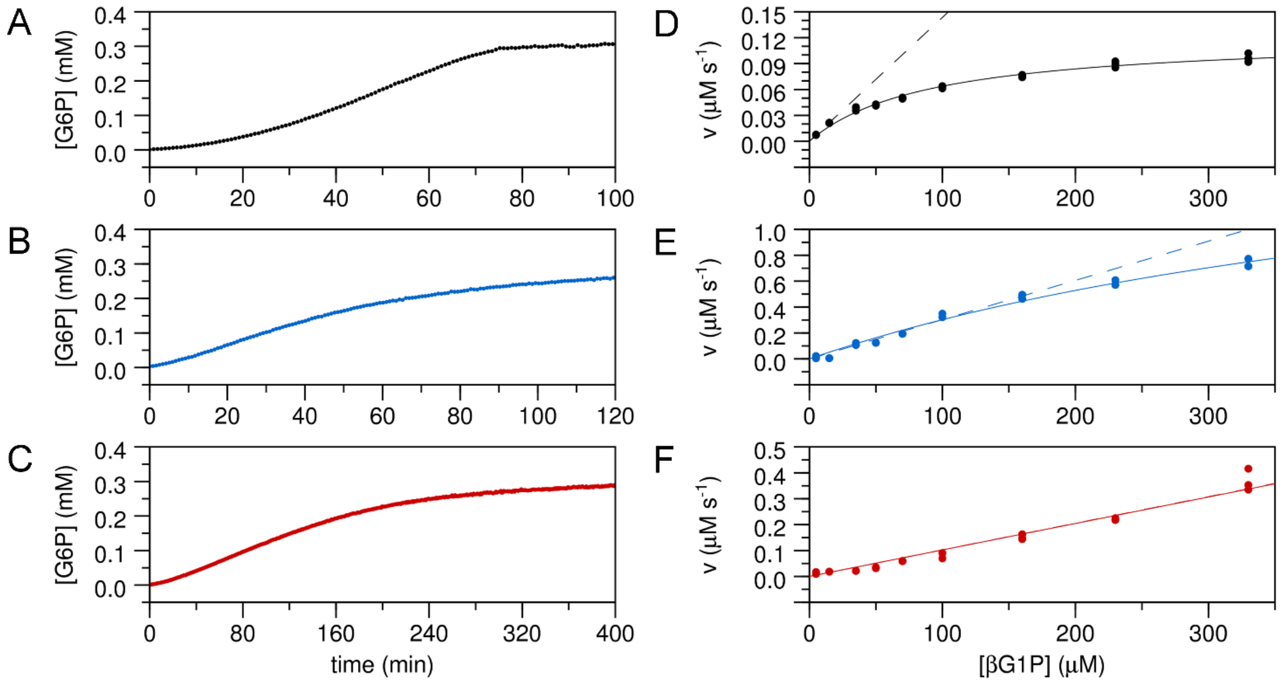

Figure S8. Reaction kinetics for the conversion of βG1P to G6P catalyzed by βPGM$_{WT}$, βPGM$_{R49K}$, and βPGM$_{R49A}$. The rate of G6P production was measured indirectly using a glucose 6-phosphate dehydrogenase coupled assay, in which G6P is oxidized and concomitant NAD$^+$ reduction is monitored by the increase in absorbance at 340 nm. (A–C) Kinetic profiles showing the time-dependent conversion of 330 µM βG1P to G6P in the presence of 20 mM AcP (10 mM AcP for βPGM$_{WT}$) in standard kinetic buffer for (A) 5 nM βPGM$_{WT}$, (B) 60 nM βPGM$_{R49K}$, and (C) 60 nM βPGM$_{R49A}$. (D–F) Michaelis-Menten plots showing the dependence of the steady-state reaction velocity (v) on βG1P concentration (5, 15, 35, 50, 70, 100, 160, 230, 330 µM) for (D) 5 nM βPGM$_{WT}$ (n=3), (E) 60 nM βPGM$_{R49K}$ (n=3), and (F) 60 nM βPGM$_{R49A}$ (n=3). Data in each plot were fitted to the standard Michaelis-Menten equation to derive apparent k_{cat} and apparent K_m (βG1P) values and the line of best fit is shown (solid lines). For βPGM$_{WT}$, the βG1P concentration range used provided reliable fitted parameters (apparent k_{cat} = 24.5 ± 0.7 s^{-1} and apparent K_m (βG1P) = 92 ± 6 µM).[38] However for βPGM$_{R49K}$, a weak βG1P affinity resulted in fitted parameters with large associated errors (apparent k_{cat} = 35 ± 5 s^{-1} and apparent K_m (βG1P) = 600 ± 100 µM). For βPGM$_{R49A}$, a linear dependence of steady-state reaction velocity on βG1P concentration precluded the derivation of fitted parameters over the accessible βG1P concentration range. Therefore, the initial data points of each Michaelis-Menten plot were fitted to a linear equation to derive the apparent k_{cat}/K_m ratio for βPGM$_{WT}$ (apparent k_{cat}/K_m = 0.29 s^{-1}·µM^{-1} for [βG1P] = 5–15 µM), βPGM$_{R49K}$ (apparent k_{cat}/K_m = 0.05 s^{-1}·µM^{-1} for [βG1P] = 5–100 µM) and βPGM$_{R49A}$ (apparent k_{cat}/K_m = 0.02 s^{-1}·µM^{-1} for [βG1P] = 5–330 µM) and the line of best fit is shown (dashed lines). For βPGM$_{R49A}$, the fitted lines derived using the standard Michaelis-Menten equation and a linear equation are overlapped.

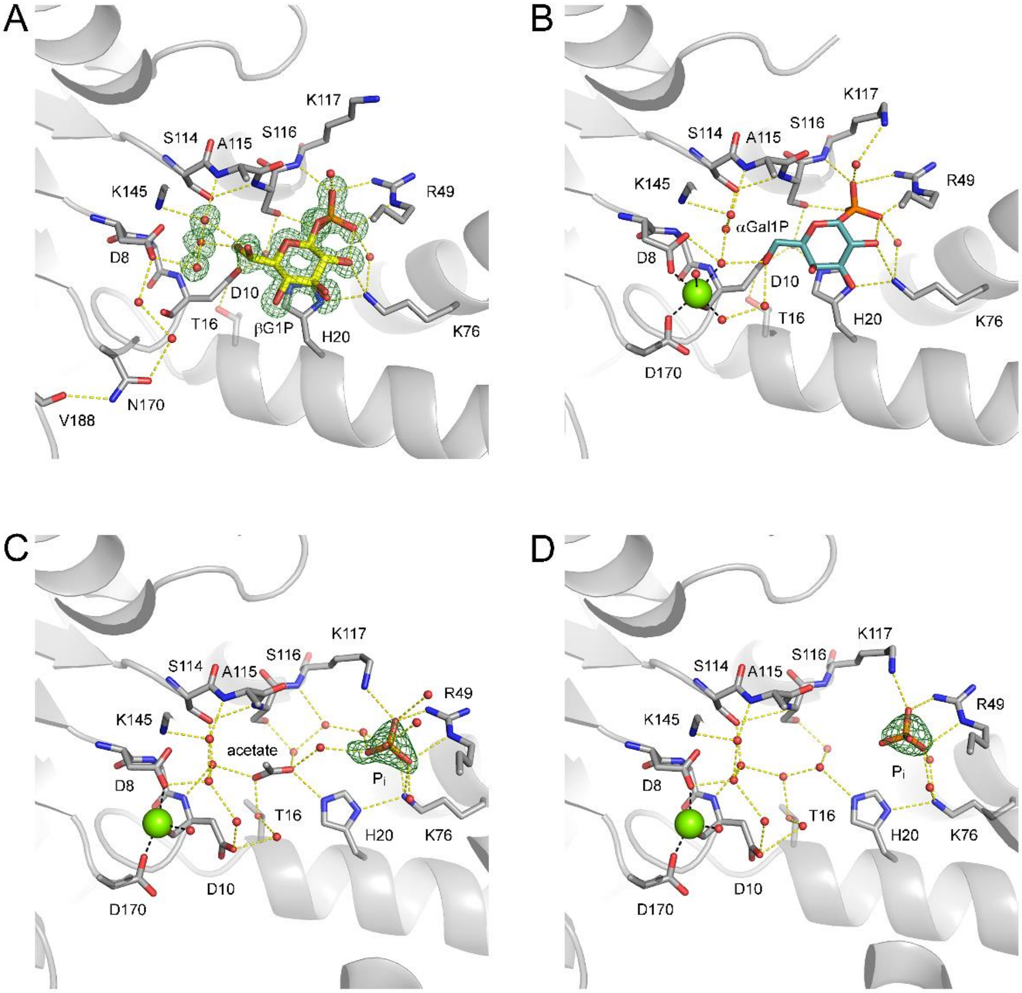

Figure S9. Difference density and active site details of the inhibited βPGM_{D170N}:βG1P complex, the βPGM_{WT}:αGal1P complex, and the βPGM_{WT}:P_i complex. The active sites of (A) inhibited βPGM_{D170N}:βG1P complex (PDB 6HDG), (B) βPGM_{WT}:αGal1P complex (PDB 1Z4O, chain A),[52] (C) βPGM_{WT}:P_i complex (PDB 6H93, chain A), and (D) βPGM_{WT}:P_i complex (PDB 6H93, chain B). Selected residues (sticks), together with βG1P (gold carbon atoms), αGal1P (teal carbon atoms), P_i occupying the distal site, structural waters (red spheres), and Mg_{cat}^{2+} (green sphere) are illustrated. Yellow dashes indicate hydrogen bonds and black dashes show metal ion coordination. Difference density (Fo – Fc, green mesh) is contoured at 3σ and was generated following ligand omission from the final structures. The 6-hydroxyl group of βG1P in the proximal site of the inhibited βPGM_{D170N}:βG1P complex has two arrangements resolved for the C5–C6 bond. The side chain of residue N118, which coordinates one of the phosphodianion oxygen atoms of both βG1P and αGal1P, has been omitted for clarity. For the βPGM_{WT}:P_i complex, two monomers are present in the asymmetric unit and analysis of the domain arrangements shows that chain B is slightly more closed.

Table S1. X-ray data collection, data processing and refinement statistics.

**Data collection and data processing statistics**					
**Complex**	**βPGM$_{D170N}$**	**βPGM$_{D170N}$:βG1P**	**βPGM$_{WT}$:P$_i$**	***β*PGM$_{R49K}$**	***β*PGM$_{R49A}$**
**PDB code**	PDB 6HDF	PDB 6HDG	PDB 6H93	PDB 6HDH	PDB 6HDI
**Wavelength (Å)**	0.92819	0.92819	0.97950	0.97624	0.97625
**Beamline, Facility**	i04-1, DLS	i04-1, DLS	i04, DLS	i03, DLS	i03, DLS
**Space group**	P2$_1$	P2$_1$2$_1$2$_1$	P2$_1$	P2$_1$	P2$_1$
**Cell dimensions:**					
**a, b, c (Å)**	38.77, 119.31, 53.17	37.56, 55.08, 105.84	38.35, 117.14, 53.19	38.13, 117.14, 53.01	38.20, 116.90, 53.17
**α, β, γ (º)**	90.0, 94.8, 90.0	90.0, 90.0, 90.0	90.0, 99.1, 90.0	90.0, 97.4, 90.0	90.0, 98.1, 90.0
**Resolution (Å)** [a]	52.98-1.40 (1.42-1.40)	48.86-1.15 (1.17-1.15)	32.05-1.77 (1.80-1.77)	31.36-1.62 (1.65-1.62)	52.64-2.03 (2.07-2.03)
Rmerge [a,b]	0.091 (1.243)	0.126 (1.986)	0.085 (1.426)	0.052 (0.770)	0.131 (0.829)
Rpim [a,c]	0.055 (0.701)	0.037 (0.634)	0.035 (0.583)	0.030 (0.487)	0.077 (0.488)
**CC-half** [a]	0.997 (0.462)	0.999 (0.562)	0.999 (0.467)	0.998 (0.491)	0.992 (0.525)
**<I/σ(I)>** [a]	8.2 (1.0)	10.8 (1.2)	12.5 (1.2)	12.7 (1.5)	7.5 (1.7)
**Completeness (%)** [a]	95.4 (95.1)	100.0 (100.0)	100.0 (100.0)	96.1 (92.7)	99.6 (99.2)
**Multiplicity** [a]	3.8 (4.0)	12.6 (10.6)	6.8 (6.9)	3.7 (3.4)	3.7 (3.8)
**Total reflections**	342050	991156	308459	210878	111221
**Unique reflections**	90031	78880	45050	56242	29676
**Molecular replacement model**	PDB 2WHE	PDB 2WF5	PDB 2WHE	PDB 2WHE	PDB 2WHE
**Refinement statistics**					
**Complex**	**βPGM$_{D170N}$**	**βPGM$_{D170N}$:βG1P**	**βPGM$_{WT}$:P$_i$**	**βPGM$_{R49K}$**	**βPGM$_{R49A}$**
**PDB code**	PDB 6HDF	PDB 6HDG	PDB 6H93	PDB 6HDH	PDB 6HDI
***R* (%)** [d] **/ *Rfree* (%)** [e]	17.2 / 22.3	14.9 / 17.8	17.9 / 23.3	18.2 / 21.8	20.5 / 27.3
**Number of atoms:**					
**Protein** [f]	1737, 1701	1772	1697, 1689	1686, 1678	1692, 1693
**Ligands** [g]	0	48	10	0	0
**Metal ions** [h]	2	1	2	2	2
**Water**	291	241	282	210	243
**Protein residues** [f]	219, 219	219	219, 219	218, 218	220, 221
**RMS deviations:**					
**Bonds (Å)**	0.01	0.01	0.12	0.01	0.01
**Angles (º)**	1.50	1.51	1.49	1.50	1.51
**Average B factors(A^2):**					
**Main chain** [f]	18.7, 18.1	12.5	26.7, 29.9	27.4	26.4, 28.3
**Side chains** [f]	23.5, 23.0	15.8	32.0, 35.1	33.4	31.5, 33.1
**Ligands** [g]	–	13.6	63.9	–	–
**Metal ions** [h]	20.4	13.5	30.5	27.7	23.3
**Water**	26.5	24.2	36.9	36.6	35.3
**Ramachandran analysis:**					
**Favored / allowed (%)**	98.2	97.8	98.4	98.6	97.7
**Disallowed (%)**	0.0	0.0	0.0	0.0	0.0
**MolProbity score (percentile)**	0.76 (100th)	0.86 (100th)	0.73 (100th)	0.97 (100th)	1.16 (100th)

[a] Values for the higher resolution shell are in parentheses.

[b] $R_{merge} = \frac{\sum_{hkl}\sum_i |I_i - I_m|}{\sum_{hkl}\sum_i I_i}$

[c] $R_{pim} = \frac{\sum_{hkl}\sqrt{\frac{1}{n-1}}\sum_{i=1}^{n}|I_i - I_m|}{\sum_{hkl}\sum_i I_i}$

where I_i and I_m are the observed intensity and mean intensity of related reflections, respectively.

[d] $R = \frac{\sum_{hkl}||F_{obs}| - k|F_{calc}||}{\sum_{hkl}|F_{obs}|}$

where F_{obs} and F_{calc} are the observed and calculated structure factor amplitudes, respectively.

Table S1 (continued). X-ray data collection, data processing and refinement statistics.

**Data collection and data processing statistics**					
**Complex**	**βPGM$_{WT}$:AlF$_4$:G6P**	**βPGM$_{R49K}$:AlF$_4$:G6P**	**βPGM$_{R49A}$:AlF$_4$:G6P**	**βPGM$_{R49K}$:MgF$_3$:G6P**	**βPGM$_{R49A}$:MgF$_3$:G6P**
**PDB code**	PDB 2WF6	PDB 6HDJ	PDB 6HDK	PDB 6HDL	PDB 6HDM
**Wavelength (Å)**	0.933	0.97625	0.97625	0.97629	0.97625
**Beamline, Facility**	ID14-2, ESRF	i03, DLS	i03, DLS	i03, DLS	i03, DLS
**Space group**	P2$_1$2$_1$2$_1$	P2$_1$2$_1$2$_1$	P2$_1$2$_1$2$_1$	P2$_1$2$_1$2$_1$	P2$_1$2$_1$2$_1$
**Cell dimensions:**					
**a, b, c (Å)**	37.80, 54.50, 105.00	104.21, 37.22, 54.22	37.23, 54.29, 104.24	37.55, 54.30, 104.20	37.30, 54.34, 104.62
**α, β, γ (°)**	90.0, 90.0, 90.0	90.0, 90.0, 90.0	90.0, 90.0, 90.0	90.0, 90.0, 90.0	90.0, 90.0, 90.0
**Resolution (Å)** [a]	20.00-1.40 (1.44-1.40)	48.10-1.16 (1.18-1.16)	54.29-1.24 (1.26-1.24)	37.55-1.16 (1.16-1.18)	54.34-1.30 (1.32-1.30)
Rmerge [a,b]	0.1 (0.37)	0.084 (1.082)	0.099 (1.019)	0.068 (1.345)	0.052 (0.263)
Rpim [a,c]	–	0.033 (0.460)	0.040 (0.480)	0.027 (0.591)	0.022 (0.138)
**CC-half** [a]	–	0.999 (0.554)	0.999 (0.530)	0.999 (0.515)	0.999 (0.944)
**<I/σ(I)>** [a]	7.3 (2.2)	11.2 (1.5)	10.1 (1.4)	14.2 (1.3)	21.1 (6.2)
**Completeness (%)** [a]	98.5 (99.4)	95.4 (88.5)	100.0 (97.9)	98.5 (92.3)	99.7 (95.3)
**Multiplicity** [a]	–	7.3 (6.2)	7.0 (5.3)	7.1 (5.9)	6.8 (4.5)
**Total reflections**	–	515051	424367	518578	361965
**Unique reflections**	43021	70516	60728	73452	53048
**Molecular replacement model**	PDB 2WF5	PDB 2WF6	PDB 2WF6	PDB 2WF5	PDB 2WF5

**Data Refinement**					
**Complex**	**βPGM$_{WT}$:AlF$_4$:G6P**	**βPGM$_{R49K}$:AlF$_4$:G6P**	**βPGM$_{R49A}$:AlF$_4$:G6P**	**βPGM$_{R49K}$:MgF$_3$:G6P**	**βPGM$_{R49A}$:MgF$_3$:G6P**
**PDB code**	PDB 2WF6	PDB 6HDJ	PDB 6HDK	PDB 6HDL	PDB 6HDM
***R* (%)** [d] **/ *Rfree* (%)** [e]	16.1 / 19.1	14.3 / 16.6	13.6 / 16.7	13.2 / 16.4	12.6 / 14.8
**Protein** [f]	1680	1739	1706	1774	1802
**Ligands** [g]	21	21	21	20	20
**Metal ions** [h]	2	1	3	2	2
**Water**	253	179	212	278	213
**Protein residues** [f]	218	219	219	219	219
**RMS deviations:**					
**Bonds (Å)**	0.01	0.01	0.01	0.01	0.01
**Angles (°)**	1.40	1.50	1.50	1.50	1.47
**Average B factors(A^2)**					
**Main chain** [f]	13.5	14.1	13.7	14.0	13.3
**Side chains** [f]	15.2	17.6	17.7	16.9	16.5
**Ligands** [g]	11.0	9.1, 8.8	9.0, 9.5	11.6, 10.9	10.0, 9.1
**Metal ions** [h]	12.4	8.6	23.1, 8.9	15.6, 9.3	14.4, 8.4
**Water**	23.4	26.1	28.4	27.5	24.1
**Ramachandran analysis**					
**Favored / allowed (%)**	96.8	98.2	97.7	97.4	98.7
**Disallowed (%)**	0.0	0.0	0.0	0.0	0.0
**MolProbity score (percentile)**	1.42 (99th)	0.76 (100th)	0.82 (100th)	1.07 (98th)	1.19 (95th)

[e] $R_{free} = \frac{\sum_{hkl \subset T} ||F_{obs}| - k|F_{calc}||}{\sum_{hkl \subset T} |F_{obs}|}$

where F_{obs} and F_{calc} are the observed and calculated structure factor amplitudes, respectively and T is the test set of data omitted from refinement (5% in this case).

[f] For structures where there are two monomers in the asymmetric unit, the values for chain A and chain B are given, respectively.

[g] Only relevant ligands are presented, other ligands (e.g. ethylene glycol and acetate, etc) have been omitted.

[h] Generally, Mg^{2+} ions were only observed in the crystals, however in some cases Na^+ ions were also noted. Where this was the case, B-factors are listed for Na^+ ions and Mg^{2+} ions, respectively.

Movie S1 (separate files). Animations comparing the active sites of the βPGM:AlF_4:G6P and βPGM:MgF_3:G6P TSA complexes. (A) Comparison between the $βPGM_{R49K}$:AlF_4:G6P complex and the $βPGM_{WT}$:AlF_4:G6P complex. (B) Comparison between the $βPGM_{R49A}$:AlF_4:G6P complex and the $βPGM_{WT}$:AlF_4:G6P complex. (C) Comparison between the $βPGM_{R49K}$:MgF_3:G6P complex and the $βPGM_{WT}$:MgF_3:G6P complex. (D) Comparison between the $βPGM_{R49A}$:MgF_3:G6P complex and the $βPGM_{WT}$:MgF_3:G6P complex.

Movie S2 (separate files). Animation illustrating part of the βPGM catalytic cycle. The animation begins with the βG1P substrate bound in the open active site of $βPGM^{P}$. Domain closure and engagement of residue D10 in the active site, allows phosphoryl transfer to occur from the aspartylphosphate group of residue D8 to βG1P, generating the βG16BP reaction intermediate and βPGM. Subsequent domain opening occurs and the steps of catalysis are reversed. (A) View showing the full βPGM enzyme. (B) View focusing on the distal site of βPGM.

Movie S3 (separate files). Animation illustrating the inhibition by hexose 1-phosphates facilitating the closure of nonphosphorylated βPGM. The animation begins with αGal1P bound in the open active site of βPGM. Domain closure and engagement of residue D10 in the active site, allows the ground state βPGM:αGal1P inhibited complex to adopt a fully closed, near-transition state conformation. Subsequent domain opening occurs with αGal1P remaining in the active site. (A) View showing the full βPGM enzyme. (B) View focusing on the distal site of βPGM.
